# TOPORS, a Dual E3 Ubiquitin and Sumo1 Ligase, Interacts with 26 S Protease Regulatory Subunit 4, Encoded by the *PSMC1* Gene

**DOI:** 10.1371/journal.pone.0148678

**Published:** 2016-02-12

**Authors:** Barbara Czub, Amna Z. Shah, Giovanna Alfano, Przemysław M. Kruczek, Christina F. Chakarova, Shomi S. Bhattacharya

**Affiliations:** 1 Institute of Ophthalmology, University College London (UCL), London EC1V 9EL, United Kingdom; 2 Centro Andaluz de Biología Molecular y Medicina Regenerativa (CABIMER), Seville-41092, Spain; Eye Hospital, Charité, GERMANY

## Abstract

The significance of the ubiquitin-proteasome system (UPS) for protein degradation has been highlighted in the context of neurodegenerative diseases, including retinal dystrophies. TOPORS, a dual E3 ubiquitin and SUMO1 ligase, forms a component of the UPS and selected substrates for its enzymatic activities, such as DJ-1/PARK7 and APOBEC2, are important for neuronal as well as retinal homeostasis, respectively. TOPORS is ubiquitously expressed, yet its mutations are only known to result in autosomal dominant retinitis pigmentosa. We performed a yeast two-hybrid (Y2H) screen of a human retinal cDNA library in order to identify interacting protein partners of TOPORS from the retina, and thus begin delineating the putative disease mechanism(s) associated with the retina-specific phenotype resulting from mutations in TOPORS. The screen led to isolation of the 26 S protease regulatory subunit 4 (P26s4/ *PSMC1*), an ATPase indispensable for correct functioning of UPS-mediated proteostasis. The interaction between endogenous TOPORS and P26s4 proteins was validated by co-immuno-precipitation from mammalian cell extracts and further characterised by immunofluorescent co-localisation studies in cell lines and retinal sections. Findings from hTERT-RPE1 and 661W cells demonstrated that TOPORS and P26s4 co-localise at the centrosome in cultured cells. Immunofluorescent staining of mouse retinae revealed a strong P26s4 reactivity at the interface between retinal pigmented epithelium (RPE) layer and the photoreceptors outer segments (OS). This finding leads us to speculate that P26s4, along with TOPORS, may have a role(s) in RPE phagocytosis, in addition to contributing to the overall photoreceptor and retinal homeostasis via the UPS.

## Introduction

The significance of the ubiquitin-proteasome system (UPS) for protein degradation has been greatly emphasised in neurodegenerative diseases [1–3]. In particular, its importance has been established in context of retinal dystrophies, highlighting the insufficient capacity of the 26 S proteasomes to degrade excess misfolded proteins as a major factor in the aetiology of photoreceptor degeneration [4–6]. TOPORS (MIM 609507), a topoisomerase I-binding, arginine/serine-rich protein, which was initially identified as a binding partner of human topoisomerase I (hTop1) and p53 in two independent yeast two-hybrid (Y2H) screens [7,8], is a component of the UPS. It acts as a dual E3 ubiquitin and SUMO1 ligase [9–11], these two ligase activities being mutually exclusive and dependent on its phosphorylation status [12,13]. Since cross-talk between the ubiquitination and SUMOylation pathways has been repeatedly demonstrated [14–17], it could be speculated that both TOPORS ubiquitination as well as SUMOylation activities affect UPS function.

E3 enzymes are a vast group of proteins mediating the final step of ubiquitination (reviewed in [18]). They convey specificity in the protein degradation processes by identifying substrates for the UPS and usually recognize only specific E2 enzyme-substrate combinations. The ubiquitin ligase activity of TOPORS is conveyed by its RING domain [10]. Interestingly, TOPORS has the capacity to recognise a greater variety of E2 ubiquitin-conjugating enzymes than most other RING E3 ligases [10,19], suggesting that it may have a more prominent regulatory role in the UPS than E3 ligases with a narrower spectrum of specificity. Several substrates for both its activities have been identified for TOPORS. Remarkably, DJ-1 (MIM 602533), implicated in early-onset Parkinson’s disease (PARK7, MIM #606324), in the aetiology of which the UPS is involved [20,21], as well as Apolipoprotein B mRNA-editing Enzyme, Catalytic Polypeptide 2 (APOBEC2) required for zebrafish retina regeneration are among the SUMOylation substrates of TOPORS [22,23], further supporting the role for the UPS in neuronal and retinal homeostasis.

Our laboratory was the first to link mutations in *TOPORS* with inherited autosomal dominant retinitis pigmentosa (adRP, locus *RP31*, MIM #609923) [24], later shown to cause approximately 1% of all adRP [25,26]. A mutation in *TOPORS* leading to an autosomal dominant pericentral retinal dystrophy (adPRD, MIM 180210) was additionally identified in a large multi-generational Scandinavian pedigree [27]. These phenotypes are of special interest since *TOPORS* is a ubiquitously expressed gene, yet its mutations have been known to result only in retinal degeneration with no other symptoms. Hence, our goal was to identify protein interacting partners of TOPORS from the human retina by performing a yeast two-hybrid (Y2H) screen.

We identified the 26 S protease regulatory subunit 4 (P26s4, UniProt #P62191), encoded by the *PSMC1* gene (MIM 602706). The 26 S proteasome comprises a barrel-shaped core catalytic particle (CP) and one or two flanking regulatory particles. The latter consist of at least twenty protein subunits constituting a base and a cap. The base includes a ring of six ATPases [28], of which P26s4 is the only one, whose ATPase activity is essential for the peptidase activity of the CP [29]. The regulatory particles confer dependence on ATP and substrate specificity.

Here we present evidence that the dual E3 ubiquitin and SUMO1 ligase, TOPORS, interacts with the 26 S protease regulatory subunit 4 (P26s4) in yeast and in mammalian cells.

## Materials and Methods

### Yeast-Two Hybrid cDNA Library Construction

The human retinal cDNA library was created in yeast using the Make Your Own “Mate & Plate™” Library System (Clontech, CA, USA) according to the manufacturer’s instructions in the pGADT7-Rec vector (Clontech, CA, USA). The library was constructed using an oligo-dT primer (OdT). The resulting preys were fused with GAL4 AD at their amino-terminus.

### Yeast Two-Hybrid Vectors

The Y2H bait construct (pBD-TOPORS: GAL4 DNA binding domain (BD) fused with full-length TOPORS) was generated using the Gateway Cloning System (Life Technologies, CA, USA) according to the manufacturer’s instructions, using vectors, which had been modified from the original Stratagene (CA, USA) Y2H vectors to contain the *att* sequences, allowing for compatibility with the Gateway Cloning System. The candidate interacting partner of TOPORS, P26s4 encoded by the *PSMC1* gene, was cloned in frame with the GAL4 activation domain (AD) using the Gateway Cloning System and the modified Stratagene vectors (pAD-PSMC1).

In-Fusion® Advantage PCR Cloning Kit (Clontech, CA, USA) was used to generate TOPORS deletion constructs, specifically pGBKT7-N-TOPORS (residues 1–380), pGBKT7-M-TOPORS (residues 373–781)and pGBKT7-C-TOPORS (residues 705–1045), for interaction characterisation in yeast.

The compatibility of the modified Stratagene and Clontech plasmid constructs was thoroughly validated prior to the Y2H screens.

### Library Screening

Matchmaker™ Yeast Two-Hybrid (Y2H) System (Clontech, CA, USA) was used following the manufacturer’s instructions. The *Saccharomyces cerevisiae* Y2H Gold strain was transformed with pBD-TOPORS and the GAL4 DNA-BD control plasmids. The *S*. *cerevisiae* Y187 strain was transformed with the human retinal cDNA library fragments and linearised pGADT7-Rec vector and the GAL4 AD control plasmids according to the manufacturer’s instructions (Yeastmaker™ Yeast Transformation System 2, Clontech, CA, USA).

The Y2H screen was performed by mating the Y2H Gold strain, expressing pBD-TOPORS, with the Y187 strain, expressing the cDNA library clones. A two-tier protein-protein interaction (PPI) selection process was used. Initially, two reporter genes, *Mel1* and *Aur1-C*, were used to detect interactions. *Mel1* encodes X-α-galactosidase, which allows for utilisation of X-α-galactose from the medium as a nutrient source. *Mel1* expression is triggered by a PPI, which is indicated by colonies excreting a blue pigment, a product of X-α-galactose utilisation. *Aur1-C* is the dominant mutant version of the *Aur1* gene, encoding an enzyme, which breaks down the highly toxic anti-fungal Aureobasidin A (AbA) drug present in the growth medium. Medium selecting for X-α-galactose utilisation and AbA resistance is referred to in the results sections as ‘Medium D.’ Positive clones were picked and re-patched on higher stringency media, referred to in the results sections as ‘Medium Q’, selecting for activation of auxotrophic markers *His3* and *Ade2* in addition to the initial *Mel1* and *Aur1-C* reporter genes (the higher stringency medium lacked histidine and adenine in addition to containing X-α-galactose and AbA).

After the second selection library plasmids were isolated from the positive clones, using the Easy Yeast Plasmid Isolation Kit (Clontech, CA, USA) and sequenced, using the The BigDye^TM^ Terminator v3.1 Cycle Sequencing Kit and the ABI PRISM® 3730 DNA Analyser (Applied Biosystems, UK). The UCSC BLAT search engine was employed to identify the inserts. The Human Genome Assembly Dec. 2013 (GRCh38/hg38) was searched, using the 'BLAT's guess' query type and the output sorting option of 'query, score'. The BLAT Search Result with the highest score was selected.

In order to confirm and characterise the interactions pAD-PSMC1 was re-transformed into yeast. PPIs between full-length TOPORS (pBD-TOPORS) or its fragments/deletion constructs (pGBKT7-N-TOPORS, pGBKT7-M-TOPORS and pGBKT7-C-TOPORS), and full-length P26s4 (pAD-PSMC1) were tested in separate experiments according to the two-tier selection processes described above. Positive (pAD-SV40 T Ag x pBD-p53) and negative (pAD-SV40 T Ag x pBD-Lamin C) interaction controls- were used throughout.

### *In Silico* Identification of Ciliary-Targeting Sequences

Several ciliary-targeting sequences (CTS) were previously described, including VxPx, RVxP, KVHPSST, AxEGG and Ax(S/A)xQ [30,31]. The PRALINE multiple sequence alignment tool (The Centre for Integrative Bioinformatics VU (IBIVU), University of Amsterdam, Netherlands) [32] was used to determine the conservation of P26s4 and TOPORS peptide sequences. Once the alignment files had been generated, the results were saved as.pdf files and the sequence was annotated manually. *In silico* identification of CTS in TOPORS (empirically confirmed to localise to centrosomes [33]) was performed as a control.

### Antibodies

Mouse monoclonal anti-TOPORS antibody (H00010210-M01) was obtained from Abnova (Taiwan); used at 1:100 for immunofluorescence (IF) and at 1:250 for Western blotting (WB). Rabbit polyclonal anti-PSMC1 (P26s4) antibody (HPA000872) was purchased from Sigma-Aldrich (MO, USA), used at 1:250 for IF and at 1:1000 for WB. Mouse (F3165) anti-FLAG antibody was purchased from Sigma-Aldrich (MO, USA) and used at 1:500. Goat polyclonal antibody against peri-centriolar material 1 (PCM1) protein (sc-50164, used at 1:250 for IF) and goat anti-V5 antibody (sc-83849, used at 1:2500) were obtained from Santa Cruz Biotechnology (TX, USA). Goat polyclonal anti-polo-like kinase 4 (PLK4) antibody (Ab2642, used at 1:150 for IF), rabbit polyclonal anti-pericentrin antibody (Ab4448, used at 1:1000) and mouse monoclonal alpha-tubulin antibody (Ab7291, used at 1:5000) were purchased from Abcam (UK). Alexa Fluor ® 488-conjugated goat anti-mouse (A11001, used at 1:400) and anti-rabbit (A11008, used at 1:400) antibodies, Alexa Fluor ® 594-conjugated goat anti-mouse (A11005, used at 1:400) and anti-rabbit (A11034, used at 1:400) antibodies, and donkey anti-goat antibodies conjugated with Alexa Fluor ® 488 (A11055, used at 1:400) and with Alexa Fluor ® A594 (A11058, used at 1:400), were purchased from Life Technologies (CA, USA). FITC-conjugated donkey anti-mouse, anti-rabbit and anti-goat IgG (1:300) and Cy ™3-conjugated donkey anti-mouse, anti-rabbit and anti-goat IgG (1:300); and horseradish peroxidase-conjugated goat anti-rabbit and anti-mouse IgG (1:10000) were purchased from Jackson Immuno Research Laboratories, Inc. (PA, USA).

### Mammalian Cell Culture

The murine 661W cone photoreceptor cell line was maintained in in Dulbecco's modified Eagle's medium (DMEM) (Life Technologies, CA, USA) supplemented with 10% FCS and penicillin–streptomycin (1000 μg/ml). Human (h)TERT-RPE1 retinal pigment epithelial and SK-N-SH neuroblastoma cell lines were maintained in Dulbecco's modified Eagle's medium (DMEM)/F-12+GlutaMAX (Life Technologies, CA, USA) supplemented with 10% FCS and penicillin–streptomycin (1000 μg/ml). All cells were grown in 6-well plates at 1.2 x10^6^ cells per well at 37°C in an atmosphere of 5% CO_2_.

### Co-Immuno-Precipitation and Western Blot Analysis

Cellular lysates were prepared from human (h)TERT-RPE1 cells and from murine 661W cells in RIPA buffer. Lysates from cellular fractions of hTERT-RPE1 cells were prepared using the ProteoExtract® Subcellular Proteome Extraction Kit (Merck Group, Germany). Prior to immuno-precipitation and SDS-PAGE/WB analysis protein concentrations were determined using the BCA Protein Assay Kit (Merck Group, Germany) and/or the Non-Interfering Protein Assay™ Kit (Merck Group, Germany).

Prior to immunoprecipitation (IP) studies SureBeads Protein G-conjugated magnetic beads (BioRad, CA, USA) were washed thoroughly and used for co-immuno-precipitation (coIP) according to the manufacturer’s instructions. Four micrograms of the mouse monoclonal anti-TOPORS antibody (Abnova) were incubated with 100 μl of beads for ten min at room temperature with rotation. The antibody-bound beads were then washed and, subsequently, incubated with 100 μl of cellular lysate (containing 0.8–1.2 mg/ml of protein) for one hour at room temperature with rotation. All concentrations and volumes are given per coIP sample. The precipitated complexes were then denatured in 1X Laemmli Sample Buffer at 75°C for ten min and loaded onto polyacrylamide gels for SDS-PAGE analysis (40 μg of cellular lysate were used per each input lane, followed by Western blotting (WB) with rabbit anti-PSMC1 (P26s4) antibody. Gradient gels (4%–20%) and PVDF membranes were used (BioRad, CA, USA). Membranes were blocked in 5% non-fat dried milk with 0.1% Tween20 in TBS. Primary and secondary antibodies were diluted as described above. ECL solution (BioRad, CA, USA) was used for visualization.

### Mammalian Expression Vectors

For characterisation in human cell lines the Gateway Cloning System (Life Technologies, CA, USA) was used to generate V5-TOPORS deletion constructs, specifically p-nV5-N-TOPORS (residues 1–380), p-nV5-M-TOPORS (residues 373–781)and p-nV5-C-TOPORS (residues 705–1045) tagged at the amino terminus. Primers, designed to generate N-, M- and C-TOPORS with *att* tails, compatible with the Gateway Cloning System, were ordered from Sigma-Aldrich (MO, USA). Full-length TOPORS, cloned into pCATCH vector tagged at the amino-terminus with FLAG, was a gift from Professor Stefan Weger (Free University of Berlin, Germany).

### Immunocytochemistry

Prior to immunofluorescence (IF) studies cells were seeded in 24-well plates on glass coverslips at 5.0 x 10^4^ cells per well and incubated for 24 h. The cells were then subjected to fixation for 20 min in 4% PFA in PBS at room temperature. They were subsequently incubated in the permeabilisation solution (4% Triton-X100 with 0.3% BSA in PBS) for five min at room temperature. The cells were washed twice in immunocytochemistry (ICC) blocking buffer (0.5% bovine serum albumin (BSA) and 20 mM glycine in PBS), followed by incubation in the blocking buffer for 15 min before staining. Cells on cover slips were incubated with the primary antibodies at room temperature overnight. Cells were washed in ICC blocking solution and incubated with secondary antibodies for 45 min at room temperature in the dark. Cells were again washed in ICC blocking solution and mounted using DAKO Fluorescence Mounting Medium (Agilent Technologies, CA, USA). Primary and secondary antibodies were diluted in concentrations described above. Nuclei were stained using 4’,6-diamidino-2-phenylindole (DAPI).

Transfections were performed in confluent hTERT-RPE1 cells using the Lipfectamine® 2000 Transfection Reagent (Thermo Fisher Scientific, MA, USA), according to the manufacturer’s instructions. The cells were fixed 24 h post transfection prior to IF staining, as described above.

### Immunohistochemistry

Eyes of C57 BLACK 6 mice were obtained from the UCL Institute of Ophthalmology Biological Resource(s) Unit (BRU) in accordance with the regulations of the Association for Research in Vision and Ophthalmology (ARVO) Statement for the Use of Animals in Ophthalmic and Vision Research. The work was approved by the UCL Institute of Ophthalmology Institutional Animal Care and Use Committee (IACUC # 70/2710).

Adult mice (6 months of age, used for all retinal studies described herein) were kept under standard housing conditions (12 hour light/dark cycle). The animals were euthanised under light conditions two to three hours after the onset of light by exposure to an increasing concentration of carbon dioxide (CO_2_) gas. Death was ensured by subsequent dislocation of the neck.

The eyes were enucleated and fixed in 4% PFA in PBS for 24 h at 4°C. Sucrose gradient infiltration was performed using: 20% sucrose for 5 h at 4°C, followed by 30% sucrose at 4°C overnight, and a solution of 15% sucrose and 50% Optical Cutting Temperature (OCT) (VWR International Ltd, UK) medium for 2 h at room temperature. The eyes were then embedded in OCT and cryo-sectioned at 10 μm. For immuno-staining retinal sections were permeabilised (0.3% Triton X-100 in PBS for ten min at room temperature) and then pre-treated with immunohistochemistry (IHC) blocking solution (5% normal goat serum (NGS) and 0.1% Triton X-100 in PBS) for 1 hour at room temperature, followed by incubation with primary antibodies. The sections were washed in 0.1% Triton X-100 in PBS and treated with appropriate secondary antibodies. Cell nuclei were stained with DAPI. Filamentous actin (F-actin) was stained with A594 conjugated-phalloidin, used at 1:400 (A12381; Life Technologies, CA, USA). Primary and secondary antibodies were diluted in concentrations described above.

### Fluorescent Microscopy Imaging

Immunostained cells and tissue cryo-sections were imaged using a ZEISS Axiovert S100 fluorescent inverted microscope and ZEISS LSM 700 laser scanning confocal microscope (Carl Zeiss, Germany). Images were processed using ZEN (blue edition) Image Browser and Adobe Photoshop CS5 (Adobe Systems, WA, USA). Deconvolution of indicated cell photomicrographs was achieved by imaging the cell in the focal plane of the centrosomal/centriolar marker first and imaging the P26s4 signal in the same plane, using the ZEISS Axiovert S100 fluorescent inverted microscope. The nucleus was then imaged in its focal plane and the three individual channels were combined manually using Adobe Photoshop CS5. This resulted is 'losing' the additional staining throughout the cell.

## Results

### TOPORS and P26s4 Interact in Yeast and Mammalian Cell Lines

The goal of this study was to identify candidate interacting partners of TOPORS from human retina in order to delineate the mechanism of retinal degeneration associated with mutations in this ubiquitously expressed gene. Over 10^7^ clones were screened (Table 1) in a two-tier selection process, utilising the *Aur1-C* PPI reporter gene at both stringency levels. Its expression results in inhibition of Aureobasidin A (AbA), which is toxic to yeast, thus reducing background growth. Firstly, the yeast culture was spread on medium supplemented with AbA and X-alpha-galactose (reporter genes *Aur1*-*C* and *Mel1*, respectively), resulting in growth of 24 colonies representing candidate interacting partners of TOPORS. Each observed colony was patched on more stringent media, which led to a successful isolation of 21 positive clones (PPIs indicated by expression of four reporter genes: *Aur1*-*C* and *Mel1* as well as auxotrophic *His3* and *Ade2*).

**Table 1:** A two-tier selection process led to identification of 21 putative interacting partners of TOPORS after the second selection level.

	Number of cDNA library clones
Total clones screened	3.29 x 10^7^
First selection level: *AUR1-C* and *MEL1* ^*1*^	24
Second selection level: *AUR1*-*C*, *MEL1*, *HIS3* and *ADE2* ^*1*^	21

^*1*^ Reporter genes expressed as a result of protein-protein interactions in the Y2H screen: *AUR1-C*, selects for resistance to AbA; *MEL1*, α-galactosidase synthesis (blue-white marker); *HIS3*, histidine synthesis (auxotrophic marker); *ADE2*, adenine synthesis (auxotrophic marker).

**Fig 1:**
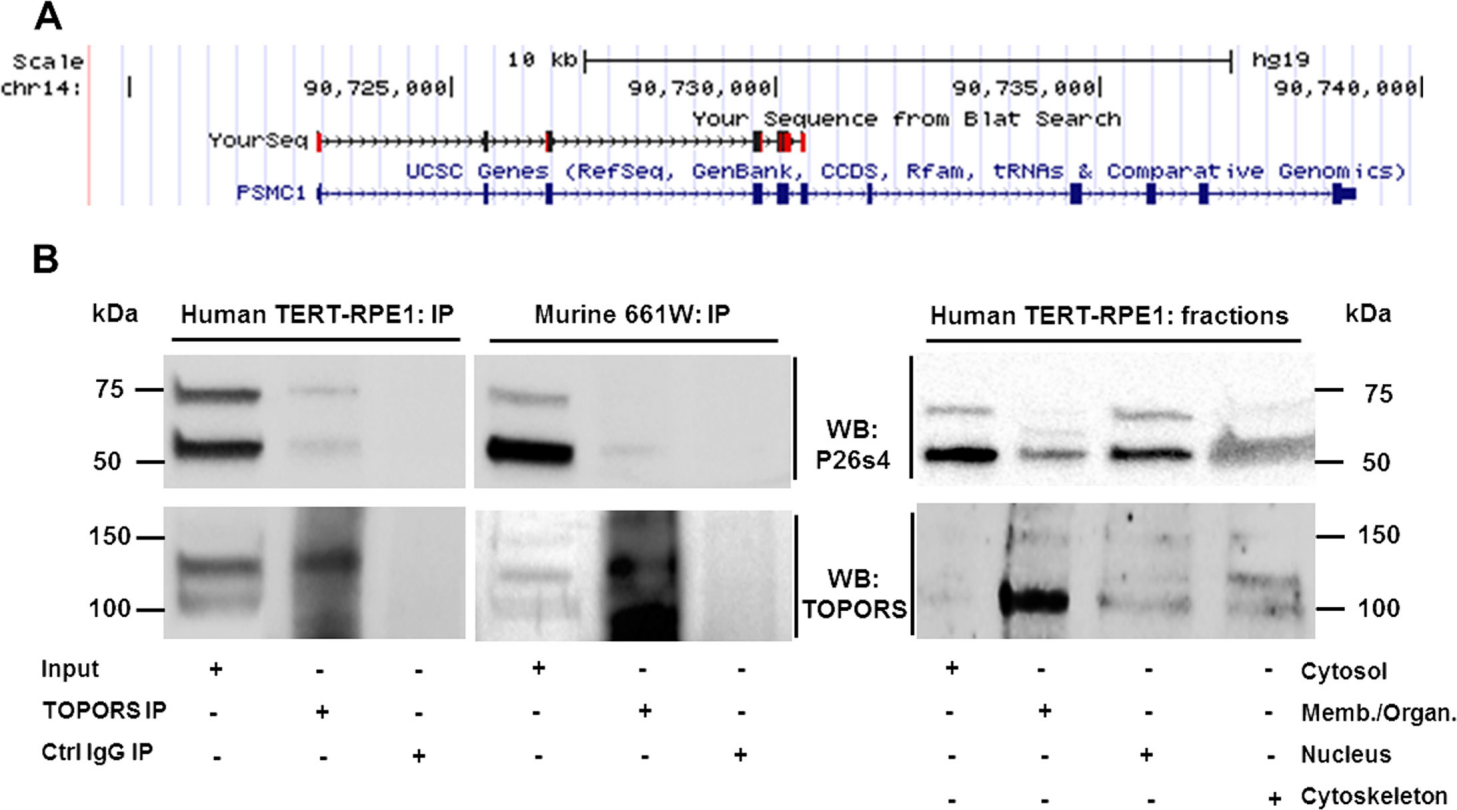
P26s4 interacts with TOPORS in yeast and mammalian cells. **A)** A *PSMC1* fragment from the human retinal library encoding P26s4 was identified as a novel interacting partner of TOPORS in the Y2H screen. The sequence alignment was generated using the UCSC Genome Browser (http://genome.ucsc.edu) on the Human Feb. 2009 (GRCh37/hg19) Assembly [34,35]. **B)** P26s4 is detected in endogenous complexes precipitated from human TERT-RPE1 and murine 661W cell lysates with an anti-TOPORS antibody. A band migrating at approximately 60 kDa was detected in total lysates and positive coIP lanes in both cell lines, indicating positive coIP of TOPORS and P26s4. An additional band migrating at approximately 75 kDa was observed in total cell lysates in both cell lines and in the coIP lane in the hTERT-RPE1 cell line. Western blotting on lysates extracted from specific cellular fractions: cytosol, membranes and organelles (Memb./Organ.), nucleus and cytoskeleton of hTERT-RPE1 cells demonstrated that the larger band is present in all tested fractions, yet is most prominent in the cytosol and the nucleus. Stripping of both membranes and re-probing for TOPORS demonstrated the protein is present in complexes precipitated from both cell lines. Re-probing to detect TOPORS in cellular fractions indicated species of different sizes are present reflecting the results from the coIP blot.

DNA was extracted from the resulting yeast patches and primers, specific to the cDNA library-vector (pGADT7-Rec), were used for amplification of cDNA inserts encoding the putative interacting proteins. The resulting PCR products were purified, sequenced and the UCSC BLAT search engine was employed to identify the inserts. One of the inserts (clone 2) was confirmed as exons 1–6 of *PSMC1* encoding the amino-terminal half of the 26 S protease regulatory subunit 4 (P26s4) (Fig 1A). These exons encode a fragment of the protein comprising an *N*-myristoylation site as well as an NADH-binding site, but they do not encode regions involved in ATP binding and hydrolysis.

The TOPORS-P26s4 interaction was subsequently validated by co-immuno-precipitation (coIP) of both endogenous proteins from human retinal pigment epithelial cells (hTERT-RPE1) and from murine 661W cone photoreceptor cell line (Fig 1B and S1 Fig). Cell extracts were precipitated using anti-TOPORS antibody, followed by Western blotting for P26s4, which generated a band of approximately 60 kDa in both cell types. In the hTERT-RPE1 cell line TOPORS also interacted with a larger P26s4 species migrating at approximately 75 kDa, which could reflect a covalent modification of P26s4, such as SUMOylation. Immunoblot analyses, performed on protein extracts from specific cellular fractions revealed that the 75 kDa is present in all cellular fractions, yet it appears most abundant in the cytosol and the nucleus. Another band unique to the fraction of membranes and organelles was additionally observed migrating approximately half-way between the 60 kDa and 75 kDa band.

### P26s4 Localises to the Centrosome

The close relationship between the 26S proteasome and the centrosome has been demonstrated in dividing HEK293 and HeLa cells [36], in granular neurons [37], and in ciliated HEK293 cells [38], as well as in the fast-growing HEK293-FT cells, in zebrafish embryos and in cilia-rich tissues, such as murine testis, kidney and retina [39]. In fact, we identified two ciliary-targeting sequences (CTS) within the peptide sequence of P26s4 (Table 2). The rhodopsin-type CTS motif (VxPx) [31] of P26s4 is located proximally to its amino-terminus. The second CTS of this protein resembles the CTS motifs found in G-protein-coupled receptors [30]; it is located approximately in the middle of the P26s4 peptide sequence. The whole protein, including its both CTS motifs, is strongly conserved among vertebrates, according to conservation scoring performed using PRALINE (The Centre for Integrative Bioinformatics VU (IBIVU), University of Amsterdam, Netherlands) [32] (data not shown). TOPORS, which is associated with the centrosome throughout the cell cycle and localises to basal body of primary cilium [33], possess two CTS motifs of the VxPx type itself, of which one (172-VTPD-175) is conserved among apes and monkeys, and the other one among vertebrates overall (484-VKPL-487), according to conservation scoring performed using PRALINE IBIVU, University of Amsterdam, Netherlands) [32] (data not shown).

**Table 2:** Ciliary-targeting sequences identified in P26s4 *in silico*.

CTS sub-type	CTS region in P26s4	Other proteins with this CTS sub-type ^2^
VxPx	52-VTPH-54 (VxPx)	Rhodopsin (P08100), polycystin-1 (P98161), polycystin-2 (Q13563)
Ax(S/A)xQ	238-AVANQ-242	G-protein-coupled receptors: Sstr3 (P32745), Htr6 (P50406), and Mchr1 (Q99705)

^2^ Reviewed in reference 31.

**Fig 2:**
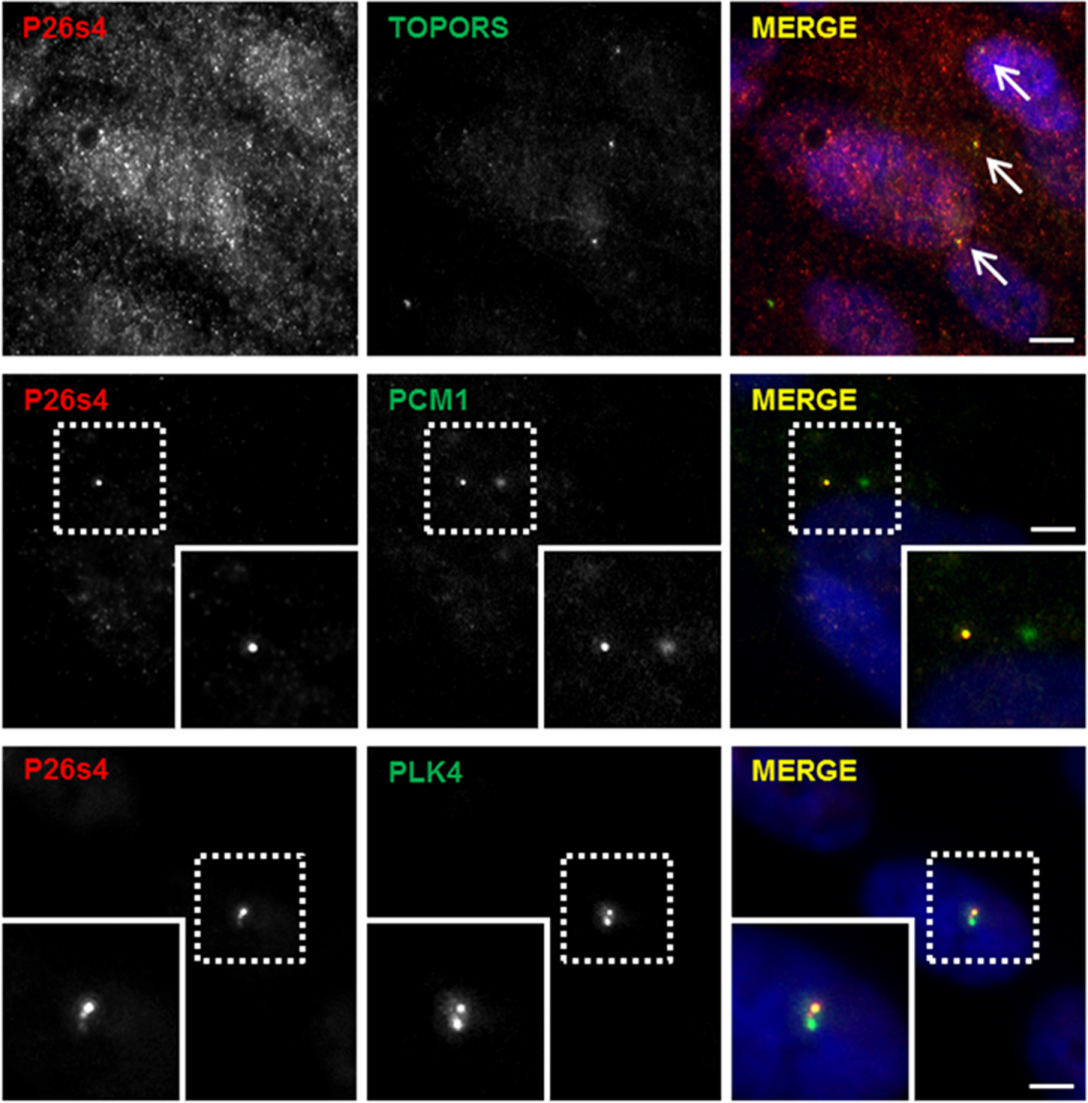
P26s4 co-localises with TOPORS and centrosomal markers in hTERT-RPE1 cells, but only at one centriole of the centrosome. P26s4 localised throughout the cell in a diffuse speckled pattern; the signal included distinct points co-localising with TOPORS staining (arrows). P26s4 co-localised with both PCM1 and PLK4 at one of the two centrioles; P26s4 signal is also observed at the linker molecules holding the two centrioles together. Scale bar: 10 μm; insets show a magnification of signals enclosed in the dashed squares. Fluorescent microscope images were taken using Zeiss Axiovert S100 inverted microscope. Images in the middle and bottom panel were deconvoluted, as described in Materials and Methods. Secondary antibody control images were collected using the same settings and generated no signals in the red and green channels.

**Fig 3:**
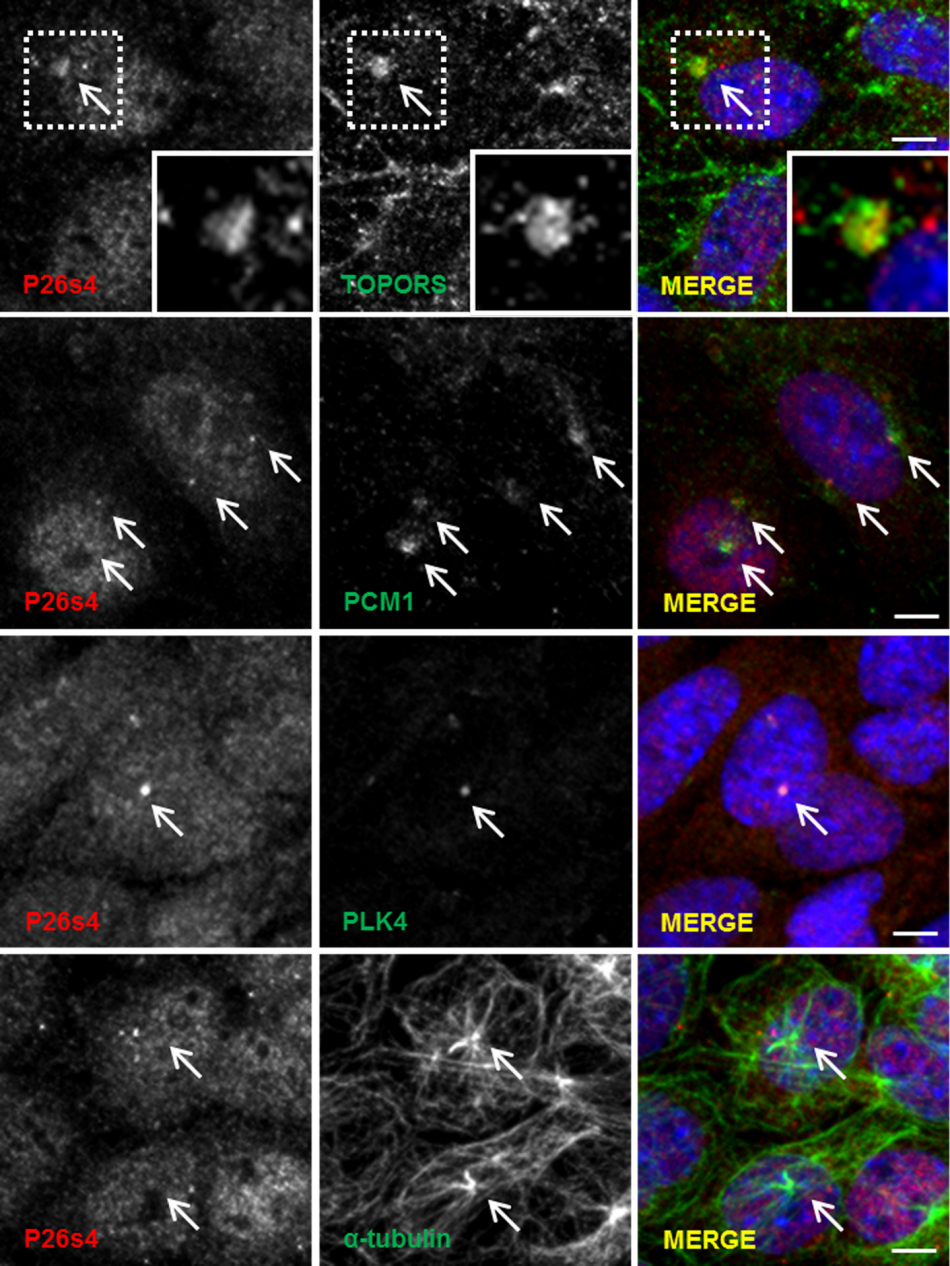
P26s4 co-localises with TOPORS and centrosomal markers in murine cone photoreceptor 661W cell line. P26s4 localised diffusely throughout the cells; the signal included distinct points co-localising with TOPORS (magnified inset), centriolar markers PCM1 and PLK4, and alpha-tubulin, where some P26s4 signal was observed at the microtubule-organising centres (centrosomes). Scale bar: 10 μm; insets show a magnification of signals enclosed in the dashed squares. Confocal microscope images were taken using Zeiss LSM 700 scanning confocal microscope. Secondary antibody control images were collected using the same settings and generated no signals in the red and green channels.

Immunofluorescence staining of hTERT-RPE1 cells (Fig 2) demonstrated diffuse, granular P26s4 signal throughout the cell, which is in agreement with the house-keeping functions of the 26 S proteasome, of which P26s4 forms a crucial component. Co-localisation with TOPORS signal occurs at several P26s4-reactive granules, which could be the centrosomes (indicated with arrows in Fig 2). Deconvoluted images showing co-staining with centrosomal markers confirmed the centrosomal-targeting of P26s4. Interestingly, it also showed that in the hTERT-RPE1 cell line this protein localises to only one centriole of the centrosome. P26s4 was not observed at the ciliary basal body in the hTERT-RPE1 cells (S2 Fig). The localisation of P26s4 was then investigated in the murine cone photoreceptor 661W cell line. The experiments revealed that P26s4 co-localised with both: TOPORS and centriolar markers in dividing (Fig 3), but not in ciliated cells (S2 Fig).

### P26s4 Associates with the C-Terminal Region of TOPORS

TOPORS is a multifunctional protein, its roles being conveyed by distinct domains located within different regions of its peptide sequence. In an attempt to separate the domains, and hence major functions, full-length TOPORS protein was divided into three portions (N-, M- and C-TOPORS; Fig 2A) to investigate, which of these domains could be responsible for mediating the interactions between TOPORS and P26s4.

N-TOPORS comprised the RING finger domain conveying the E3 ubiquitin ligase activity and phosphoserine 98 involved in up-regulation of TOPORS ubiquitination activity [12]. M-TOPORS contained an SR/RS dipeptide region, which includes a region required for interaction with SUMO1 as well as a SUMO1 acceptor site at lysine 560 [40]. Serine 718 was also included; its phosphorylation leads to up-regulation of the ubiquitin ligase activity whilst down-regulating the SUMO1 ligase activity of TOPORS [13]. C-TOPORS included the *RP31* mutational hotspot region [24–26] and fragments required for interaction with SUMO1 as well as its E2 conjugating enzyme, UBC9. Phosphoserine 718 was also included within C-TOPORS [40].

**Fig 4:**
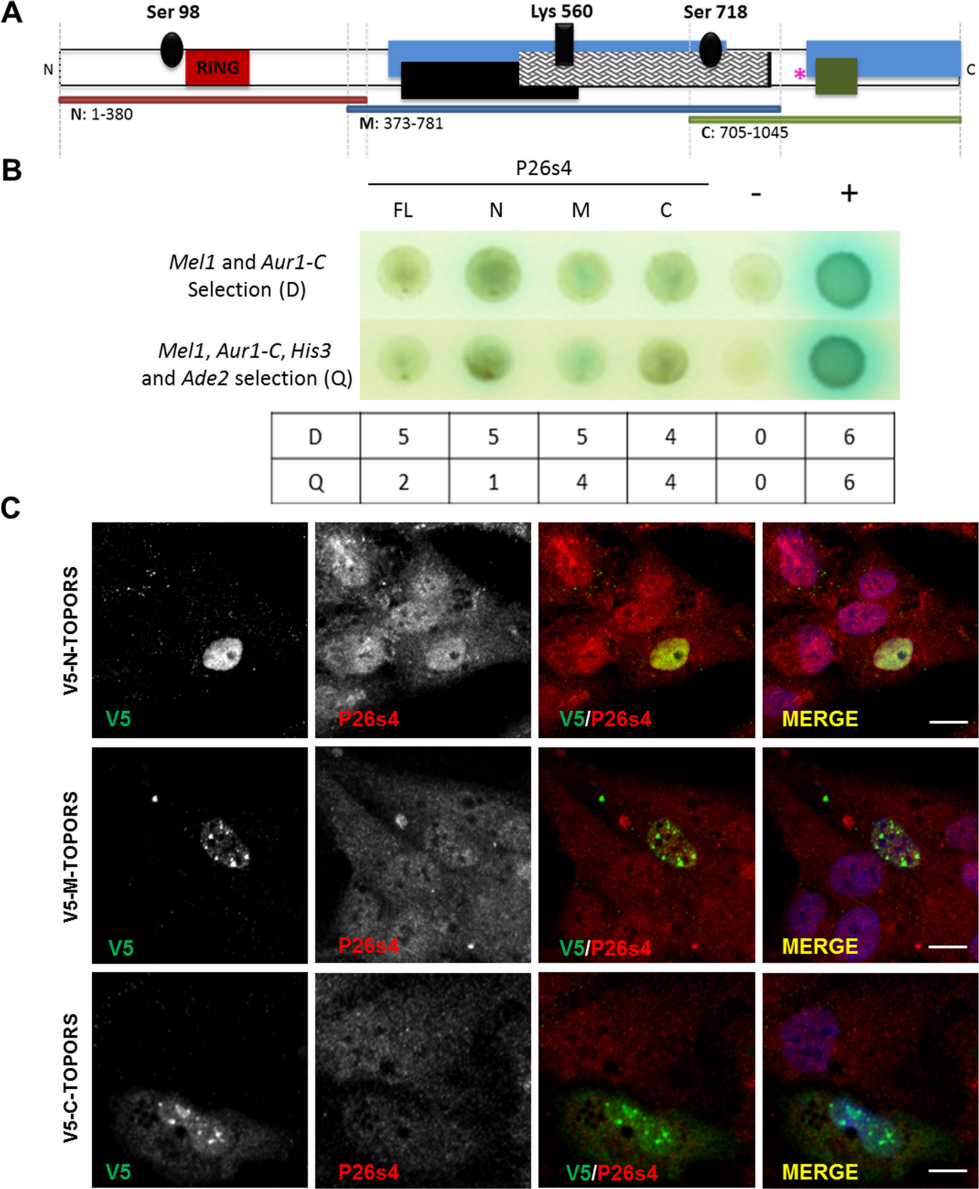
P26s4 interacts with TOPORS in yeast and in human cell lines. **A)** TOPORS protein domain diagram. Black ovals denote known phosphorylation sites at serines 98 and 718 required for regulation of E3 ligase activities of TOPORS; dark red box (residues 103–141) indicates the RING domain; dark cross-hatched box (residues 530–777) represents the arginine- and serine-rich (SR/RS) domain; a SUMO1 acceptor site at Lys 560 with a covalently bound SUMO1 modification (perpendicular black box) is indicated within the SR/RS domain; blue boxes (residues 415–737 and 854–1045) indicate regions required for interaction with SUMO1; black box (residues 437–574) represents a fragment required for SUMOylation of TOPORS at Lys 560; the green box (aa: 871–917) represents a region required for minimal interaction with UBC9. The pink asterisk represents the *RP31* mutational hotspot. The horizontal red, blue and green bars represent TOPORS deletion constructs used in experiments presented in panel B. Diagram not to scale. **B)** Direct PPI between TOPORS, its deletion constructs and P26s4 tested by Y2H. Each experiment was performed six times; interactions recorded in a minimum of four out of the six experiments were interpreted as an overall positive result. The figure panel depicts a representative raw result from one of the six experiments. The bottom table includes a summary of results from all six experiments; total number of positive PPI results at a lower (D), or higher (Q) stringency level, are indicated for each tested PPI pair. Key: FL, full-length TOPORS (residues 1–1045); N, N-TOPORS; M, M-TOPORS; C, C-TOPORS. Positive PPI control (‘+’): AD-SV40 T Ag x BD-p53. Negative PPI control (‘–‘): AD-SV40 T Ag x BD-Lamin C; BD, GAL4 DNA Binding Domain; AD, GAL4 Activation Domain. D, medium selecting for X-α-galactose utilisation (blue colonies) and AbA resistance, i.e. activation of two PPI reporter genes; Q, medium selecting for X-α-galactose utilisation (blue colonies), AbA resistance, histidine synthesis and adenine synthesis, i.e. activation of four PPI reporter genes. **C)** P26s4 co-localised with all V5-tagged artificial TOPORS fragments at nuclei of hTERT-RPE1 cells, and it additionally co-localised with the V5-tagged C-TOPORS fragment in the cytoplasm of hTERT-RPE1 cells transfected with V5-tagged TOPORS deletion constructs, and co-stained for V5 and P26s4.

The deletion constructs (Fig 2A) were tested directly in yeast for interactions with full-length P26s4. The findings demonstrated that P26s4 readily interacted with constructs M and C (Table in Fig 4B), which are associated with the E3 SUMO1 ligase activity of TOPORS. The interaction between M-TOPORS and P26s4 resulted in activation of two reporter genes in five out of six experiments at the lower stringency, and all four reporter genes were activated in five out of six experiments at the higher stringency. C-TOPORS interacted with P26s4 in four out of six experiments at both stringency levels. The association between N-TOPORS and P26s4 was weaker in comparison, resulting in consistent activation of two reporter genes only, whereas growth on medium selecting for activation of all four interaction reporter genes was observed only in one experiment. These findings indicate that P26s4 may be a SUMOylation substrate for TOPORS.

We subsequently investigated the localisation of the artificial TOPORS fragments in hTERT-RPE1 cells. Following transfection with the V5-tagged deletion constructs and co-staining with P26s4 we found that each one of the TOPORS fragments co-localised with P26s4 within different regions of the cells (Fig 4C), which is in agreement with the ubiquitous nature of both proteins. Co-transfection with the FLAG-tagged full-length TOPORS and co-staining with TOPORS and pericentrin additionally revealed that the N-TOPORS construct was observed in the nucleus and at the centrosome (S3 Fig), the M-TOPORS construct only in the nucleus (S4 Fig), whereas the C-TOPORS was present in both the nucleus and cytoplasm (S5 Fig).

In summary, findings from experiments using TOPORS deletion constructs revealed that P26s4 associates strongly with the M- and C-TOPORS fragments (Fig 4B), which are associated with the SUMOylation activities of TOPORS. When expressed in hTERT-RPE1 cells, the M- and C-TOPORS fragments localise to the nucleus (S4 Fig), as well as nucleus and cytoplasm (S5 Fig), respectively. This is in agreement with our findings from immunoblotting experiments on cellular fractions (Fig 1B) demonstrating that the larger (approximately 75 kDa) species of P26s4, which could be covalently modified, e.g. by SUMO1, is present predominantly in the cytosol and the nucleus.

### P26s4 Co-Localises with TOPORS at Distal Outer Segments and the RPE

Although studies in yeast and mammalian cells can provide some degree of confirmation towards the interaction in question, to begin evaluating the biological significance and relevance of the identified interactions to the original research question, it is important to determine where the proteins localise in the retina.

**Fig 5:**
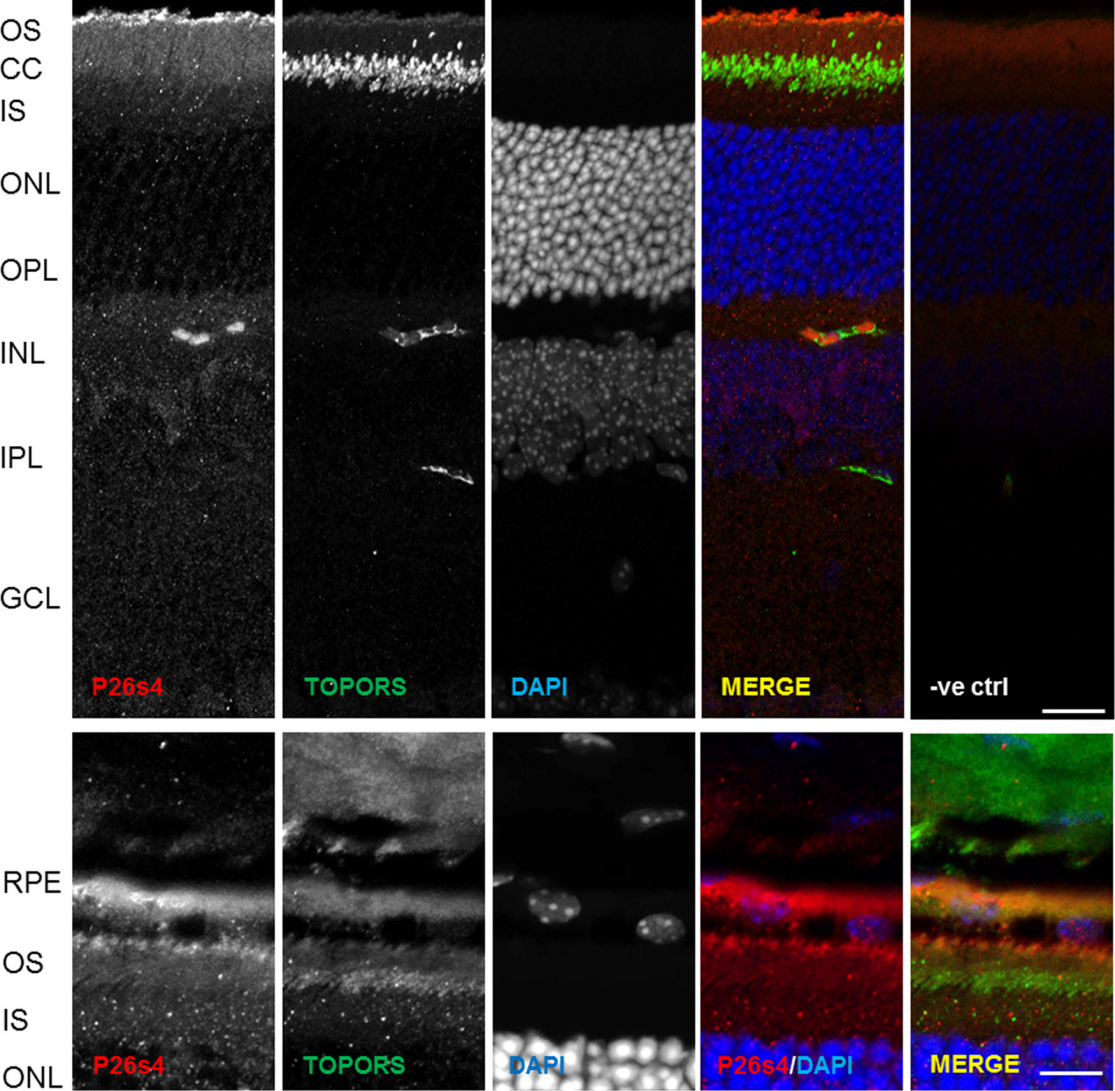
P26s4 localises to distal photoreceptor outer segments (OS) and retinal pigment epithelium (RPE) in murine retina sections. P26s4 localises diffusely throughout the tissue with distinct signal observed at the distal OS regions of photoreceptor cells, whereas TOPORS localises predominantly to the connecting cilia (top panel: scale bar 40 μm). The strongest P26s4 signal is observed at the RPE-OS interface and the RPE (bottom panel: scale bar 20 μm). RPE, retinal pigment epithelium; OS, rod photoreceptor outer segment; CC, connecting cilium; IS, rod photoreceptor inner segment; ONL, outer nuclear layer; OPL, outer plexiform layer; INL, inner nuclear layer; IPL, inner plexiform layer; GCL, ganglion cell layer; -ve ctrl, negative control, a retinal cryo-section immunostained only with DAPI and secondary antibodies. Nuclei stained using DAPI. Confocal microscope images were taken using the Zeiss LSM 700. Secondary antibody control images were collected using the same settings and generated no signals in the red and green channels.

Immunostaining of the adult mouse retina revealed a P26s4 signal very specifically localised at the interface between the photoreceptor outer segment (OS) and the RPE layer (Fig 5 and S6 Fig) in light-adapted retinal sections obtained from animals sacrificed 2 to 3 h after the onset of light. TOPORS signal co-localises with P26s4 in this retinal region (Fig 5 and S7 Fig) in addition to localising at the photoreceptor connecting cilium as previously demonstrated [33]. It was previously shown that OS phagocytosis by the RPE peaks at 2 h after light onset [41,42], as well as that P26s4 is involved in protein degradation processes not associated with its regulatory role within the 26 S proteasome complex [43]. Thus, the co-localisation of P26s4 and TOPORS at the tips of the OS and/or the RPE could indicate that, given their known roles in proteostasis, these two proteins may be involved in recycling of the tips of the OS.

The findings indicate that P26s4 could be involved in OS phagocytosis by the RPE, since the protein’s signal localises specifically to the OS-RPE interface at the peak of RPE phagocytic activity.

## Discussion

*PSMC1* is a ubiquitously expressed gene, encoding an essential 26 S protease regulatory subunit 4 (P26s4). It appears to have no explicit link to the retina, according to literature published to date. However, it is often purposefully knocked down in a conditional manner to induce neurodegeneration in mouse models [2,44,45] and in humans it has been linked to spinocerebellar ataxia type 7 (MIM 602706 and MIM #164500). Our findings, which demonstrate a protein-protein interaction (PPI) between P26s4 and TOPORS (Fig 1 and S1 Fig), implicated in retinitis pigmentosa, support the involvement of P26s4 in maintenance of the retina with a potential role in retinal degeneration.

The PPIs between full-length TOPORS and P26s4, demonstrated in mammalian cell lines, indicated that at least two P26s4 species migrating on SDS-PAGE gels at approximately 60 kDa and 75 kDa are expressed at the protein level. Both species co-precipitated with TOPORS from hTERT-RPE1 cells, whereas only the smaller species was detected in protein complexes precipitated with TOPORS from the 661W cells. Further analysis on cellular fractionation extracts revealed that the two species are differentially expressed between cellular fractions (Fig 1B). Subsequently, experiments were performed to delineate the domains of TOPORS, which mediate its interactions with this newly-identified protein partner. Findings showed that P26s4 interacted most strongly with M-TOPORS and C-TOPORS constructs (Fig 4B). Both of these TOPORS constructs comprise regions involved in its SUMOylation activities and, additionally, C-TOPORS also includes the *RP31* mutational hotspot. These results suggest that TOPORS may be important for SUMOylation of P26s4, which could in turn regulate the protein degradation processes. The resulting impairments in proteostasis could help to explain the phenotype associated with *RP31* mutations, as photoreceptors are much more vulnerable to perturbations than less specialised cell types. *In silico* analysis indicates that at least one SUMO-interacting motif (SIM: ΨKxE, where Ψ is a hydrophobic amino acid, and x is any amino acid [46]) is found within human P26s4 sequence, at residues 63LKLE66. It is possible that residues 174MKVE177 could act as another SIM, however, methionine is only weakly hydrophobic, making this region less likely to be involved in SUMO binding. Both motifs are located within the amino-terminal half of P26s4 protein, which was pulled out from the Y2H screen, as an interacting partner of TOPORS (Fig 1A).

TOPORS is associated with the centrosome throughout the cell cycle, localising to mitotic centrioles and to basal bodies of primary cilia, including connecting cilia of photoreceptor cells [33]. The involvement of P26s4/*PSMC1* in cell cycle defects has been documented in mouse embryonic fibroblasts [47]. Hence, we were interested in evaluating whether the interaction between TOPORS and P26s4 could occur at the centrosome and/or the basal body of photoreceptor connecting cilium. Initial immunostaining experiments of hTERT-RPE1 (Fig 2) and 661W (Fig 3) cells revealed that P26s4 does indeed co-localise with one centriole in dividing hTERT-RPE1 cells and both centrioles in dividing 661W cells. However, no localisation at ciliary basal body was observed in either hTERT-RPE1 cells (S2 Fig), or the 661W cells (data not shown).

Immunoblotting for P26s4 in protein extracts isolated from different fractions of non-synchronised hTERT-RPE1 cells revealed that the protein is abundant in both the nuclear and cytosolic fractions (Fig 1B). The P26s4 protein contains a consensus N-myristoylation sequence (residues 1–6), conserved in humans, *Saccharomyces cerevisiae* (yeast), *Drosophila melanogaster* (fruit-fly), and in *Arabidopsis thaliana* (mouse-ear cress) [48,49]. N-myristoylation of P26s4 is required for localisation of the proteasomal complex to the nucleus, potentially by anchoring it within the nuclear envelope. Mutations of glycine 2, otherwise modified by the myristoyl attachment, trigger re-location of the proteasome from the nucleus to the cytoplasm [48]. Furthermore, the M-TOPORS and C-TOPORS constructs, which associated most strongly with P26s4 in yeast (Fig 4B), localise to the nucleus (M-TOPORS, S4 Fig), and the nucleus and cytoplasm (C-TOPORS, S5 Fig). The N-TOPORS fragment, which localised to the nucleus and centrioles (S4 Fig), associated less strongly with P26s4 (Fig 4B). Therefore, collectively these results indicate the centrosome is probably not a major site of interaction between TOPORS and P26s4.

Alternatively, TOPORS SUMOylation of P26s4 may modulate its localisation, or it may act as a pre-requisite for other post-translational modifications of P26s4. It was previously shown in zebrafish that SUMOylation of APOBEC2, with the involvement of TOPORS E3 SUMO1 ligase activity, was required for determining its subcellular localisation [23]. Furthermore, SUMOylation of DJ1/PARK7, another substrate of TOPORS E3 SUMO1 ligase activity, is also required for its translocation from cytoplasm to the nucleus [50]. Thus, TOPORS could similarly be involved in SUMOylation of P26s4 to mediate its translocation between the two sub-cellular regions. This role would be especially important in non-dividing cells, such as neurons, including photoreceptor cells. At the molecular level neurodegeneration is often characterised by neuronal inclusions of protein aggregates, e.g. the neuronal intranuclear inclusion disease (NIID; MIM 603472) [51]. It should be highlighted that, whereas in dividing cells nuclear protein aggregates can often be cleared out by autophagy, when the nuclear envelope dissociates during cell division, terminally differentiated neurons can only rely on intranuclear UPS since their nuclear membrane never dissociates.

A similar homeostasis mechanism could apply to the primary cilium, which is commonly referred to as a ‘privileged membrane domain,’ protected from the surrounding cytosolic contents by the transition zone, forming a ciliary diffusion barrier, or the ciliary necklace [52]. This barrier comprises nuclear pore-like structures within the ciliary necklace restricting access to the intraciliary compartment [53,54]. It is possible that the intraciliary space is subject to similar stresses (protein misfolding and aggregation) as the intranuclear space, and cilia-targeted proteins may similarly rely on SUMOylation, or another covalent modification, in addition to CTS motifs, for their translocation into the cilium. Future work should address these important points in context of photoreceptor outer segments (OS), which are highly specialised sensory primary cilia. Whereas typical primary cilia which serve as cellular antennae detecting chemical or mechanical stimuli, photoreceptor OS are highly specialised primary cilia, which detect photons of light [55]. As with neuronal nuclei, the OS never dissociate. Therefore, although they are regularly turned over by RPE phagocytosis at their distant ends, their proximal regions may as well need to rely on the UPS for homeostasis.

The most distinct P26s4 immuno-reactivity signal in murine retinae was observed at the OS-RPE interface, in retinal sections from mice euthanised 2 h after light onset, which correlates with the greatest RPE phagocytosis activity [41,42]. A previously unreported, weak TOPORS signal was also detected in this region (Fig 5 and S7 Fig), which is the site of OS phagocytosis by the RPE. Furthermore, diffuse P26s4 signal was additionally detected throughout the inner retina (Fig 5) in much the same diffuse appearance as observed in cell lines (Figs 2, 3 and 4C). Unlike TOPORS, P26s4 was not observed at the centriolar basal bodies of photoreceptor connecting cilia (Fig 5). This was not unexpected based on findings from cellular localisation studies, which demonstrated that P26s4 was associated with the centriole in dividing (Figs 2 and 3), but not ciliated (S2 Fig) cells. On the contrary, TOPORS is associated with the centrioles throughout all cell cycle phases [33], including the phase of quiescence (G0), during which cells do not divide and become ciliated, and the mother centriole forms the ciliary basal body [56]. Thus, the localisation of P26s4 and TOPORS in photoreceptors (Fig 5), which are quiescent cells, reflects our findings from the cellular studies.

We propose that P26s4 is important for photoreceptor homeostasis by participating in both RPE phagocytosis as well as protein degradation by the UPS. This could explain the co-localisation of TOPORS and P26s4 at the interface between the photoreceptor OS and the RPE (Fig 5 and S6 and S7 Figs). Yet, even though the OS are regularly turned over by RPE phagocytosis at their distal ends, the region proximal to the connecting cilium must rely on another protein degradation pathway to maintain correct functioning of this highly active cellular system, such as the UPS. If this latter mechanism is impaired, this may lead to intraciliary stress in photoreceptor cells due to protein misfolding, analogous to that observed in NIID, and, subsequently, photoreceptor cell death. Our findings support such a role for P26s4 and/or the UPS in retinal maintenance and degeneration.

## Supporting Information

S1 Fig P26s4 interacts with TOPORS in mammalian cells. Full-sized images of blots shown in Fig 1 are presented. P26s4 is detected in endogenous complexes precipitated from human TERT-RPE1 and murine 661W cell lysates with an anti-TOPORS antibody. A band migrating at approximately 60 kDa was detected in total lysates and positive coIP lanes in both cell lines, indicating positive coIP of TOPORS and P26s4. An additional band migrating at approximately 75 kDa was observed in total cell lysates in both cell lines and in the coIP lane in the hTERT-RPE1 cell line. Western blotting on lysates extracted from specific cellular fractions: cytosol, membranes and organelles (Memb./Organ.), nucleus and cytoskeleton of hTERT-RPE1 cells demonstrated that the larger band is present in all tested fractions, but is most prominent in the cytosol and the nucleus. Stripping of both membranes and re-probing for TOPORS demonstrated the protein is present in complexes precipitated from both cell lines. Re-probing to detect TOPORS in cellular fractions indicated species of different sizes are present reflecting the results from the coIP blot.
(TIF)

S2 Fig P26s4 did not co-localise with primary cilia (marked with α-tubulin, inset) in ciliated hTERT-RPE1 cells; however, it co-localised with α-tubulin at a punctate localization, where no primary cilium was observed (indicated by arrow, upper panel), likely representing the centrosome. Scale bar: 10 μm. Deconvoluted images from ZEISS Axiovert S100 fluorescent inverted microscope.
(TIF)

S3 Fig N-TOPORS localises to the nucleus and centrioles. Human (h)TERT-RPE1 cells were either co-transfected with V5-tagged N-TOPORS and FLAG-tagged full-length TOPORS (top panel), or with V5-tagged N-TOPORS only (middle and bottom panels). The V5-tagged artificial N-TOPORS fragment co-localised with FLAG-tagged full-length TOPORS (top panel) and endogenous TOPORS (middle panel) at nuclei of hTERT-RPE1 cells and it co-localised with pericentrin at the centrioles.
(TIF)

S4 Fig M-TOPORS localises to the nucleus. Human (h)TERT-RPE1 cells were either co-transfected with V5-tagged M-TOPORS and FLAG-tagged full-length TOPORS (top panel), or with V5-tagged M-TOPORS only (middle and bottom panels). The V5-tagged artificial M-TOPORS fragment co-localised with FLAG-tagged full-length TOPORS (top panel) and endogenous TOPORS (middle panel) at nuclei of hTERT-RPE1 cells, but it did not co-localise with pericentrin.
(TIF)

S5 Fig C-TOPORS localises to the nucleus and cytoplasm. Human (h)TERT-RPE1 cells were either co-transfected with V5-tagged C-TOPORS and FLAG-tagged full-length TOPORS (top panel), or with V5-tagged C-TOPORS only (middle and bottom panels). The V5-tagged artificial C-TOPORS fragment co-localised with FLAG-tagged full-length TOPORS (top panel) at nuclei of hTERT-RPE1 cells and it co-localised with endogenous TOPORS at the nucleus (middle panel), however it did not co-localise with pericentrin.
(TIF)

S6 Fig P26s4 localises to distal photoreceptor outer segments (OS) and retinal pigment epithelium (RPE) in murine retina sections. P26s4 localises diffusely throughout the photoreceptor OS with distinct P26s4 signal being observed at the RPE-OS interface and the RPE (both panels). Phalloidin staining for filamentous actin was used as a marker delineating OS structure (bottom panel). RPE, retinal pigment epithelium; OS, rod photoreceptor outer segment; IS, rod photoreceptor inner segment. Nuclei stained using DAPI. Scale bar 20 μm. Confocal microscope images were taken using the Zeiss LSM 700. Secondary antibody control images were collected using the same settings and generated no signals in the red and green channels.
(TIF)

S7 Fig TOPORS localises throughout the retina with the strongest signal observed at the photoreceptor connecting cilium, and also a distinct signal at the distal region of the outer segments (OS). OS, photoreceptor outer segment; CC, connecting cilium; IS, photoreceptor inner segment; ONL, outer nuclear layer; OPL, outer plexiform layer; INL, inner nuclear layer; IPL, inner plexiform layer. Nuclei stained using DAPI. Scale bar 20 μm. Confocal microscope images were taken using the Zeiss LSM 700. Secondary antibody control images were collected using the same settings and generated no signals in the red and green channels.
(TIF)

## References

[1] Ardley HC, Hung CC, Robinson PA (2005) The aggravating role of the ubiquitin-proteasome system in neurodegeneration. FEBS Lett 579: 571–576. 15670810 10.1016/j.febslet.2004.12.058

[2] Bedford L, Hay D, Devoy A, Paine S, Powe DG, et al (2008) Depletion of 26S proteasomes in mouse brain neurons causes neurodegeneration and Lewy-like inclusions resembling human pale bodies. J Neurosci 28: 8189–8198. 10.1523/JNEUROSCI.2218-08.2008 18701681 PMC6670564

[3] Pandey UB, Nie Z, Batlevi Y, McCray BA, Ritson GP, et al (2007) HDAC6 rescues neurodegeneration and provides an essential link between autophagy and the UPS. Nature 447: 859–863. 17568747 10.1038/nature05853

[4] Illing ME, Rajan RS, Bence NF, Kopito RR (2002) A rhodopsin mutant linked to autosomal dominant retinitis pigmentosa is prone to aggregate and interacts with the ubiquitin proteasome system. J Biol Chem 277: 34150–34160. 12091393 10.1074/jbc.M204955200

[5] Lobanova ES, Finkelstein S, Skiba NP, Arshavsky VY (2013) Proteasome overload is a common stress factor in multiple forms of inherited retinal degeneration. Proc Natl Acad Sci U S A 110: 9986–9991. 10.1073/pnas.1305521110 23716657 PMC3683722

[6] Campello L, Esteve-Rudd J, Cuenca N, Martín-Nieto J (2013) The ubiquitin-proteasome system in retinal health and disease. Mol Neurobiol 47: 790–810. 10.1007/s12035-012-8391-5 23339020

[7] Haluska P, Saleem A, Rasheed Z, Ahmed F, Su EW, et al (1999) Interaction between human topoisomerase I and a novel RING finger/arginine-serine protein. Nucleic Acids Res 27: 2538–2544. 10352183 10.1093/nar/27.12.2538 PMC148458

[8] Zhou R, Wen H, Ao SZ (1999) Identification of a novel gene encoding a p53-associated protein. Gene 235: 93–101. 10415337 10.1016/s0378-1119(99)00203-6

[9] Pungaliya P, Kulkarni D, Park HJ, Marshall H, Zheng H, et al (2007) TOPORS functions as a SUMO-1 E3 ligase for chromatin-modifying proteins. J Proteome Res 6: 3918–3923. 17803295 10.1021/pr0703674

[10] Rajendra R, Malegaonkar D, Pungaliya P, Marshall H, Rasheed Z, et al (2004) Topors functions as an E3 ubiquitin ligase with specific E2 enzymes and ubiquitinates p53. J Biol Chem 279: 36440–36444. 15247280 10.1074/jbc.C400300200

[11] Weger S, Hammer E, Heilbronn R (2005) Topors acts as a SUMO-1 E3 ligase for p53 in vitro and in vivo. FEBS Lett 579: 5007–5012. 16122737 10.1016/j.febslet.2005.07.088

[12] Park HJ, Zheng H, Kulkarni D, Kerrigan J, Pungaliya P, et al (2008) Identification of phosphorylation sites of TOPORS and a role for serine 98 in the regulation of ubiquitin but not SUMO E3 ligase activity. Biochemistry 47: 13887–13896. 10.1021/bi801904q 19053840

[13] Yang X, Li H, Zhou Z, Wang WH, Deng A, et al (2009) Plk1-mediated phosphorylation of Topors regulates p53 stability. J Biol Chem 284: 18588–18592. 10.1074/jbc.C109.001560 19473992 PMC2707202

[14] Denuc A, Marfany G (2010) SUMO and ubiquitin paths converge. Biochem Soc Trans 38: 34–39. 10.1042/BST0380034 20074031

[15] Miteva M, Keusekotten K, Hofmann K, Praefcke GJ, Dohmen RJ (2010) Sumoylation as a signal for polyubiquitylation and proteasomal degradation. Subcell Biochem 54: 195–214. 10.1007/978-1-4419-6676-6_16 21222284

[16] Perry JJ, Tainer JA, Boddy MN (2008) A SIM-ultaneous role for SUMO and ubiquitin. Trends Biochem Sci 33: 201–208. 10.1016/j.tibs.2008.02.001 18403209

[17] Wilson VG, Heaton PR (2008) Ubiquitin proteolytic system: focus on SUMO. Expert Rev Proteomics 5: 121–135. 10.1586/14789450.5.1.121 18282128 PMC3467698

[18] Welchman RL, Gordon C, Mayer RJ (2005) Ubiquitin and ubiquitin-like proteins as multifunctional signals. Nat Rev Mol Cell Biol 6: 599–609. 16064136 10.1038/nrm1700

[19] Ying M, Huang X, Zhao H, Wu Y, Wan F, et al (2011) Comprehensively surveying structure and function of RING domains from Drosophila melanogaster. PLoS One 6: e23863 10.1371/journal.pone.0023863 21912646 PMC3166285

[20] McNaught KS, Olanow CW, Halliwell B, Isacson O, Jenner P (2001) Failure of the ubiquitin-proteasome system in Parkinson's disease. Nat Rev Neurosci 2: 589–594. 11484002 10.1038/35086067

[21] Martins-Branco D, Esteves AR, Santos D, Arduino DM, Swerdlow RH, et al (2012) Ubiquitin proteasome system in Parkinson's disease: a keeper or a witness? Exp Neurol 238: 89–99. 10.1016/j.expneurol.2012.08.008 22921536 PMC4077023

[22] Shinbo Y, Taira T, Niki T, Iguchi-Ariga SM, Ariga H (2005) DJ-1 restores p53 transcription activity inhibited by Topors/p53BP3. Int J Oncol 26: 641–648. 15703819

[23] Powell C, Cornblath E, Goldman D (2014) Zinc-binding domain-dependent, deaminase-independent actions of apolipoprotein B mRNA-editing enzyme, catalytic polypeptide 2 (Apobec2), mediate its effect on zebrafish retina regeneration. J Biol Chem 289: 28924–28941. 10.1074/jbc.M114.603043 25190811 PMC4200251

[24] Chakarova CF, Papaioannou MG, Khanna H, Lopez I, Waseem N, et al (2007) Mutations in TOPORS cause autosomal dominant retinitis pigmentosa with perivascular retinal pigment epithelium atrophy. Am J Hum Genet 81: 1098–1103. 17924349 10.1086/521953 PMC2265653

[25] Bowne SJ, Sullivan LS, Gire AI, Birch DG, Hughbanks-Wheaton D, et al (2008) Mutations in the TOPORS gene cause 1% of autosomal dominant retinitis pigmentosa. Mol Vis 14: 922–927. 18509552 PMC2391085

[26] Sullivan LS, Bowne SJ, Reeves MJ, Blain D, Goetz K, et al (2013) Prevalence of Mutations in eyeGENE(R) Probands with a Diagnosis of Autosomal Dominant Retinitis Pigmentosa. Invest Ophthalmol Vis Sci. 10.1167/iovs.13-12605 PMC3778873 23950152

[27] Selmer KK, Grøndahl J, Riise R, Brandal K, Braaten O, et al (2010) Autosomal dominant pericentral retinal dystrophy caused by a novel missense mutation in the TOPORS gene. Acta Ophthalmol 88: 323–328. 10.1111/j.1755-3768.2008.01465.x 19183411

[28] Tanahashi N, Suzuki M, Fujiwara T, Takahashi E, Shimbara N, et al (1998) Chromosomal localization and immunological analysis of a family of human 26S proteasomal ATPases. Biochem Biophys Res Commun 243: 229–232. 9473509 10.1006/bbrc.1997.7892

[29] Voges D, Zwickl P, Baumeister W (1999) The 26S proteasome: a molecular machine designed for controlled proteolysis. Annu Rev Biochem 68: 1015–1068. 10872471 10.1146/annurev.biochem.68.1.1015

[30] Hsiao YC, Tuz K, Ferland RJ (2012) Trafficking in and to the primary cilium. Cilia 1: 4 10.1186/2046-2530-1-4 23351793 PMC3541539

[31] Mazelova J, Astuto-Gribble L, Inoue H, Tam BM, Schonteich E, et al (2009) Ciliary targeting motif VxPx directs assembly of a trafficking module through Arf4. EMBO J 28: 183–192. 10.1038/emboj.2008.267 19153612 PMC2637330

[32] Simossis V, Kleinjung J, Heringa J (2003) An overview of multiple sequence alignment. Curr Protoc Bioinformatics Chapter 3: Unit 3.7. 10.1002/0471250953.bi0307s03 18428699

[33] Chakarova CF, Khanna H, Shah AZ, Patil SB, Sedmak T, et al (2011) TOPORS, implicated in retinal degeneration, is a cilia-centrosomal protein. Hum Mol Genet 20: 975–987. 10.1093/hmg/ddq543 21159800 PMC3033188

[34] Kent WJ (2002) BLAT—the BLAST-like alignment tool. Genome Res 12: 656–664. 11932250 10.1101/gr.229202 PMC187518

[35] Kent WJ, Sugnet CW, Furey TS, Roskin KM, Pringle TH, et al (2002) The human genome browser at UCSC. Genome Res 12: 996–1006. 12045153 10.1101/gr.229102 PMC186604

[36] Wigley WC, Fabunmi RP, Lee MG, Marino CR, Muallem S, et al (1999) Dynamic association of proteasomal machinery with the centrosome. J Cell Biol 145: 481–490. 10225950 10.1083/jcb.145.3.481 PMC2185077

[37] Puram SV, Kim AH, Park HY, Anckar J, Bonni A (2013) The ubiquitin receptor S5a/Rpn10 links centrosomal proteasomes with dendrite development in the mammalian brain. Cell Rep 4: 19–30. 10.1016/j.celrep.2013.06.006 23831032 PMC4103748

[38] Gerdes JM, Liu Y, Zaghloul NA, Leitch CC, Lawson SS, et al (2007) Disruption of the basal body compromises proteasomal function and perturbs intracellular Wnt response. Nat Genet 39: 1350–1360. 17906624 10.1038/ng.2007.12

[39] Liu YP, Tsai IC, Morleo M, Oh EC, Leitch CC, et al (2014) Ciliopathy proteins regulate paracrine signaling by modulating proteasomal degradation of mediators. J Clin Invest 124: 2059–2070. 24691443 10.1172/JCI71898 PMC4001542

[40] Weger S, Hammer E, Engstler M (2003) The DNA topoisomerase I binding protein topors as a novel cellular target for SUMO-1 modification: characterization of domains necessary for subcellular localization and sumolation. Exp Cell Res 290: 13–27. 14516784 10.1016/s0014-4827(03)00292-1

[41] Tosini G, Iuvone PM, McMahon DG, Collin SP (2014) The retina and circadian rhythms New York: Springer viii, 238 pages p.

[42] Anderson RE, LaVail MM, Hollyfield JG (2010) Retinal degenerative diseases: laboratory and therapeutic investigations New York; London: Springer xlvii, 714 p. p.

[43] Lipson C, Alalouf G, Bajorek M, Rabinovich E, Atir-Lande A, et al (2008) A proteasomal ATPase contributes to dislocation of endoplasmic reticulum-associated degradation (ERAD) substrates. J Biol Chem 283: 7166–7175. 10.1074/jbc.M705893200 18174173

[44] Gómez-Garre P, Jesús S, Carrillo F, Cáceres-Redondo MT, Bernal-Bernal I, et al (2012) PSMC1 Gene in Parkinson's Disease. Eur Neurol 68: 193–198. 10.1159/000339003 22948515

[45] Paine SM, Anderson G, Bedford K, Lawler K, Mayer RJ, et al (2013) Pale body-like inclusion formation and neurodegeneration following depletion of 26S proteasomes in mouse brain neurones are independent of α-synuclein. PLoS One 8: e54711 10.1371/journal.pone.0054711 23382946 PMC3559752

[46] Sampson DA, Wang M, Matunis MJ (2001) The small ubiquitin-like modifier-1 (SUMO-1) consensus sequence mediates Ubc9 binding and is essential for SUMO-1 modification. J Biol Chem 276: 21664–21669. 11259410 10.1074/jbc.M100006200

[47] Rezvani N, Elkharaz J, Lawler K, Mee M, Mayer RJ, et al (2012) Heterozygosity for the proteasomal Psmc1 ATPase is insufficient to cause neuropathology in mouse brain, but causes cell cycle defects in mouse embryonic fibroblasts. Neurosci Lett 521: 130–135. 10.1016/j.neulet.2012.05.070 22677101

[48] Kimura A, Kato Y, Hirano H (2012) N-myristoylation of the Rpt2 subunit regulates intracellular localization of the yeast 26S proteasome. Biochemistry 51: 8856–8866. 10.1021/bi3007862 23102099

[49] Lee KH, Marshall RS, Slivicke LM, Vierstra RD (2012) Genetic analyses of the Arabidopsis 26S proteasome regulatory particle reveal its importance during light stress and a specific role for the N-terminus of RPT2 in development. Plant Signal Behav 7: 973–978. 10.4161/psb.20934 22836496 PMC3474698

[50] Kato I, Maita H, Takahashi-Niki K, Saito Y, Noguchi N, et al (2013) Oxidized DJ-1 inhibits p53 by sequestering p53 from promoters in a DNA-binding affinity-dependent manner. Mol Cell Biol 33: 340–359. 10.1128/MCB.01350-12 23149933 PMC3554126

[51] Lieberman AP, Robitaille Y, Trojanowski JQ, Dickson DW, Fischbeck KH (1998) Polyglutamine-containing aggregates in neuronal intranuclear inclusion disease. Lancet 351: 884 9525376 10.1016/S0140-6736(05)70296-8

[52] Chih B, Liu P, Chinn Y, Chalouni C, Komuves LG, et al (2012) A ciliopathy complex at the transition zone protects the cilia as a privileged membrane domain. Nat Cell Biol 14: 61–72. 10.1038/ncb2410 22179047

[53] Kee HL, Verhey KJ (2013) Molecular connections between nuclear and ciliary import processes. Cilia 2: 11 10.1186/2046-2530-2-11 23985042 PMC3765448

[54] Nozawa YI, Lin C, Chuang PT (2013) Hedgehog signaling from the primary cilium to the nucleus: an emerging picture of ciliary localization, trafficking and transduction. Curr Opin Genet Dev 23: 429–437. 10.1016/j.gde.2013.04.008 23725801 PMC3913210

[55] Rachel RA, Li T, Swaroop A (2012) Photoreceptor sensory cilia and ciliopathies: focus on CEP290, RPGR and their interacting proteins. Cilia 1: 22 10.1186/2046-2530-1-22 23351659 PMC3563624

[56] Bettencourt-Dias M, Carvalho-Santos Z (2008) Double life of centrioles: CP110 in the spotlight. Trends Cell Biol 18: 8–11. 18068367 10.1016/j.tcb.2007.11.002

