# Supplementary figures and images for: TOPORS, a Dual E3 Ubiquitin and Sumo1 Ligase, Interacts with 26 S Protease Regulatory Subunit 4, Encoded by the PSMC1 Gene
Source: PLoS One. 2016 Feb 12;11(2):e0148678. doi: 10.1371/journal.pone.0148678 (PMC4752349; doi:10.1371/journal.pone.0148678)

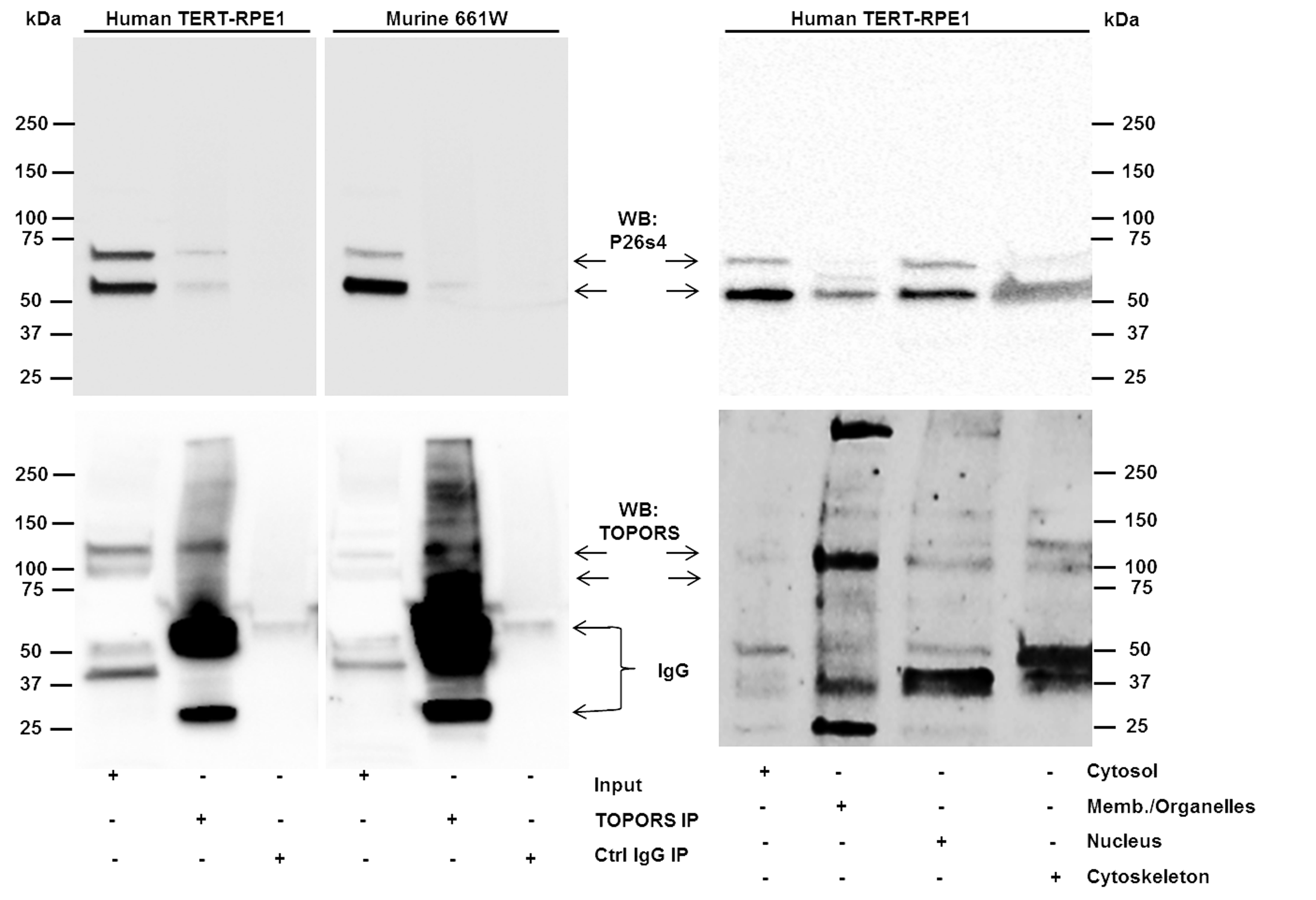

Supplement: S1 Fig — Full-sized images of blots shown in Fig 1 are presented. P26s4 is detected in endogenous complexes precipitated from human TERT-RPE1 and murine 661W cell lysates with an anti-TOPORS antibody. A band migrating at approximately 60 kDa was detected in total lysates and positive coIP lanes in both cell lines, indicating positive coIP of TOPORS and P26s4. An additional band migrating at approximately 75 kDa was observed in total cell lysates in both cell lines and in the coIP lane in the hTERT-RPE1 cell line. Western blotting on lysates extracted from specific cellular fractions: cytosol, membranes and organelles (Memb./Organ.), nucleus and cytoskeleton of hTERT-RPE1 cells demonstrated that the larger band is present in all tested fractions, but is most prominent in the cytosol and the nucleus. Stripping of both membranes and re-probing for TOPORS demonstrated the protein is present in complexes precipitated from both cell lines. Re-probing to detect TOPORS in cellular fractions indicated species of different sizes are present reflecting the results from the coIP blot. (TIF) [file pone.0148678.s001.tif]

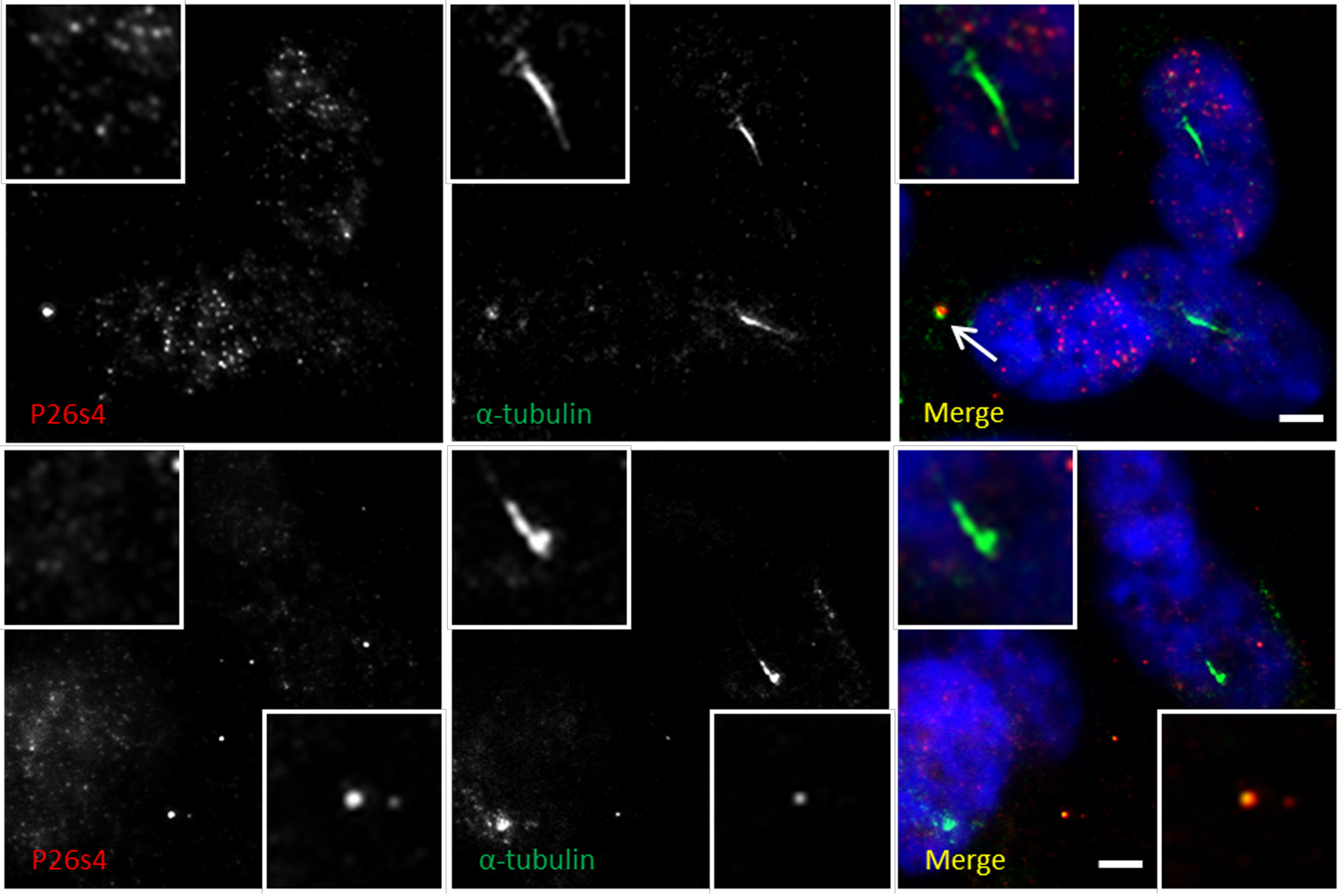

Supplement: S2 Fig — Scale bar: 10 μm. Deconvoluted images from ZEISS Axiovert S100 fluorescent inverted microscope. (TIF) [file pone.0148678.s002.tif]

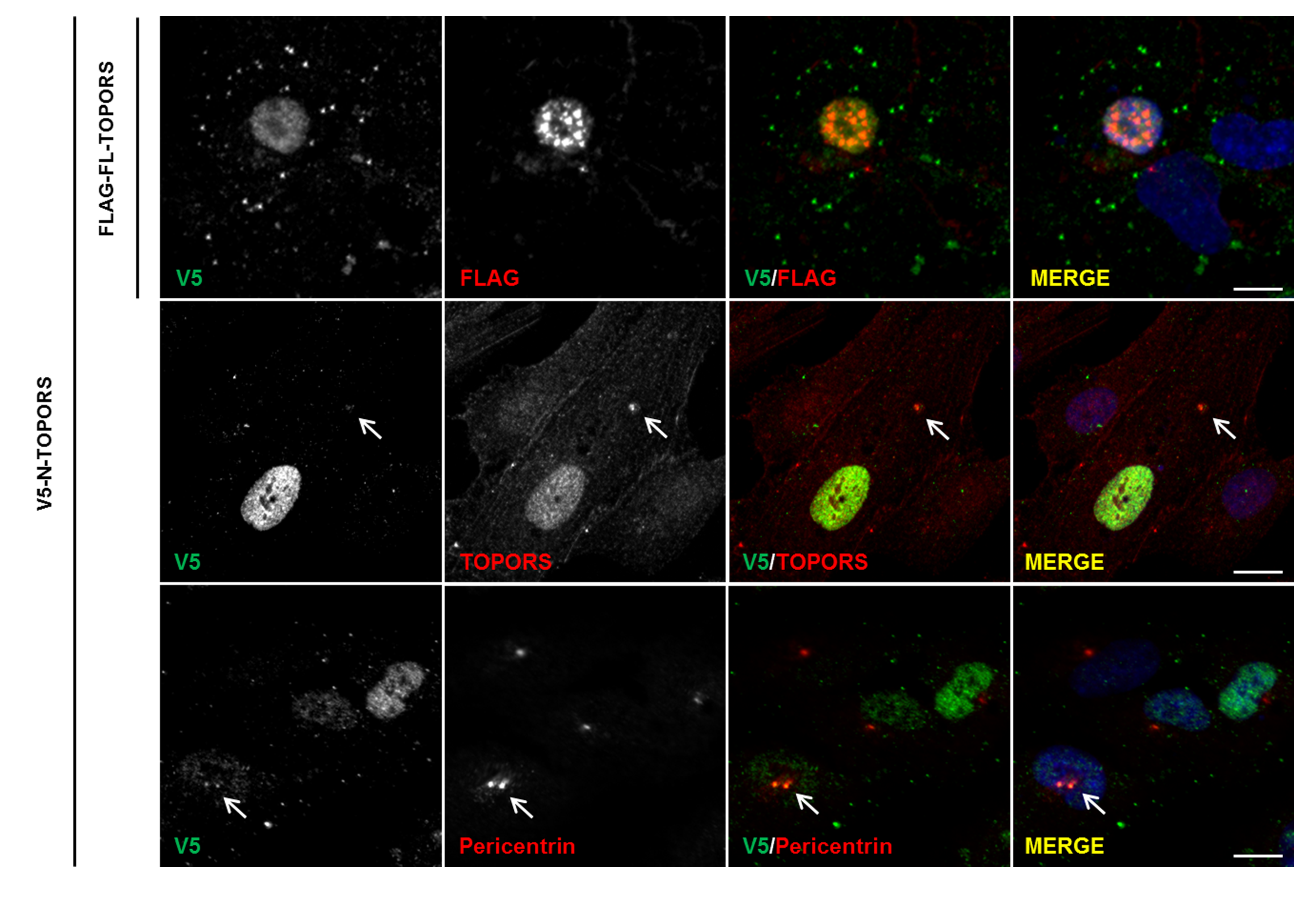

Supplement: S3 Fig — Human (h)TERT-RPE1 cells were either co-transfected with V5-tagged N-TOPORS and FLAG-tagged full-length TOPORS (top panel), or with V5-tagged N-TOPORS only (middle and bottom panels). The V5-tagged artificial N-TOPORS fragment co-localised with FLAG-tagged full-length TOPORS (top panel) and endogenous TOPORS (middle panel) at nuclei of hTERT-RPE1 cells and it co-localised with pericentrin at the centrioles. (TIF) [file pone.0148678.s003.tif]

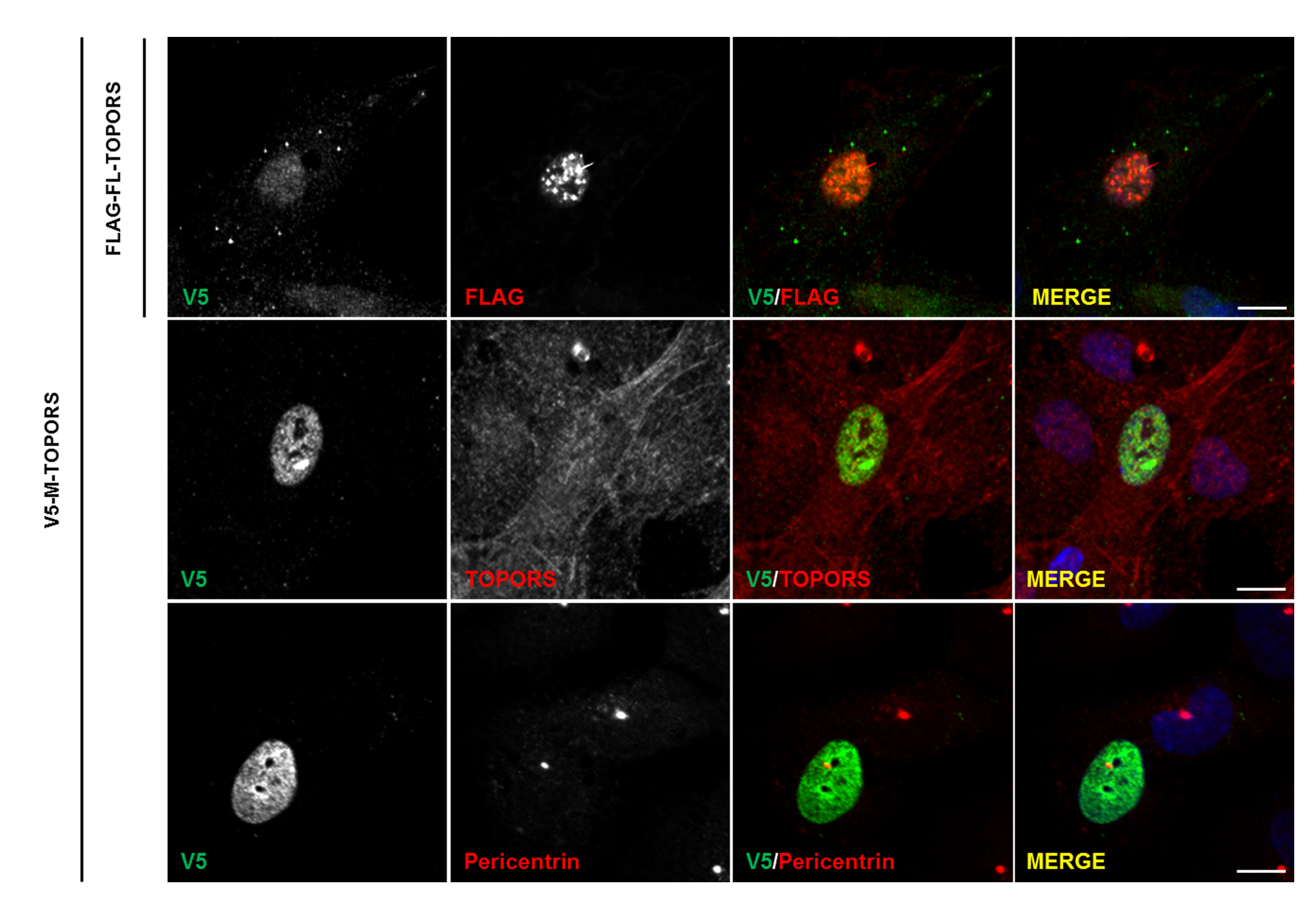

Supplement: S4 Fig — Human (h)TERT-RPE1 cells were either co-transfected with V5-tagged M-TOPORS and FLAG-tagged full-length TOPORS (top panel), or with V5-tagged M-TOPORS only (middle and bottom panels). The V5-tagged artificial M-TOPORS fragment co-localised with FLAG-tagged full-length TOPORS (top panel) and endogenous TOPORS (middle panel) at nuclei of hTERT-RPE1 cells, but it did not co-localise with pericentrin. (TIF) [file pone.0148678.s004.tif]

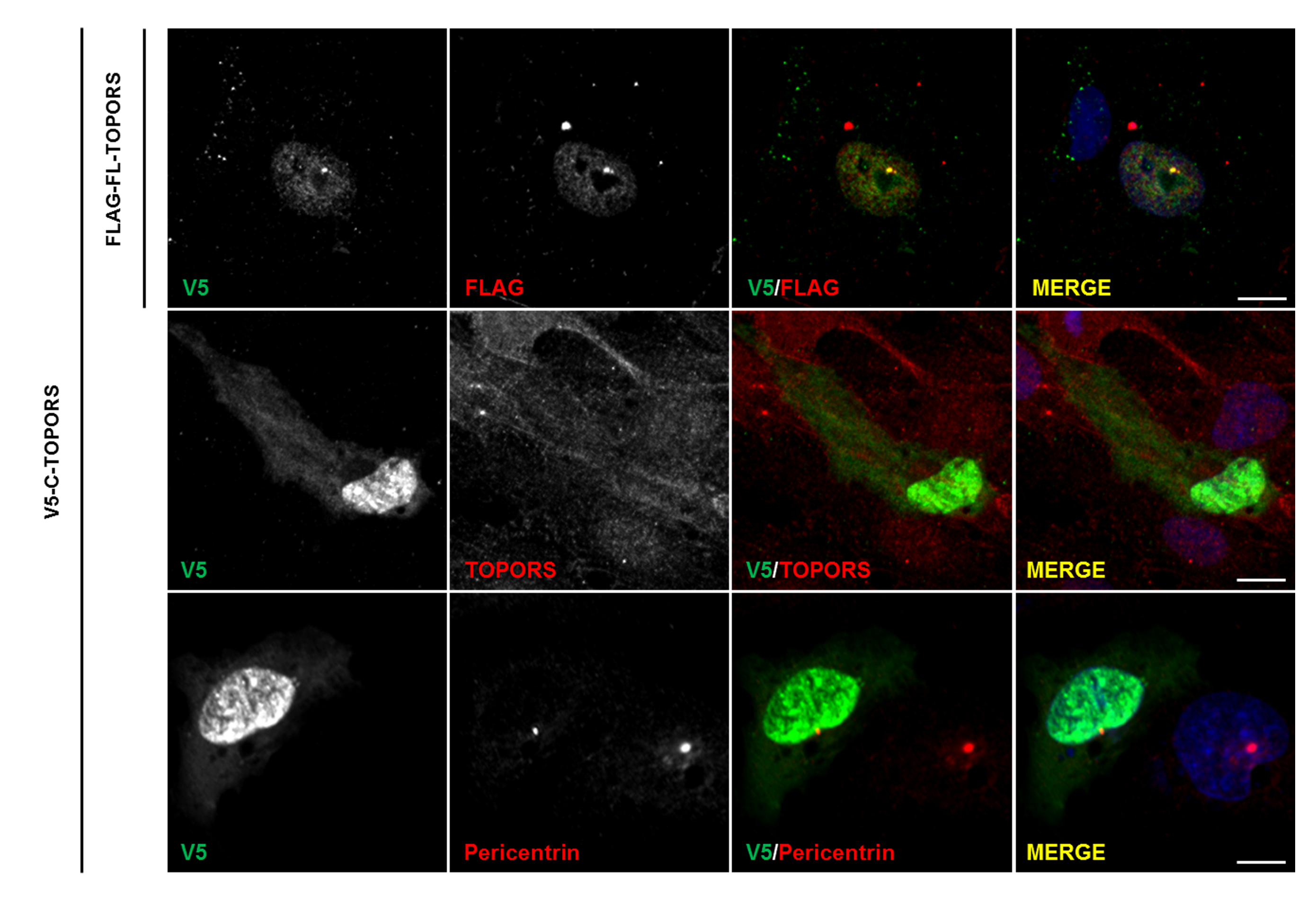

Supplement: S5 Fig — Human (h)TERT-RPE1 cells were either co-transfected with V5-tagged C-TOPORS and FLAG-tagged full-length TOPORS (top panel), or with V5-tagged C-TOPORS only (middle and bottom panels). The V5-tagged artificial C-TOPORS fragment co-localised with FLAG-tagged full-length TOPORS (top panel) at nuclei of hTERT-RPE1 cells and it co-localised with endogenous TOPORS at the nucleus (middle panel), however it did not co-localise with pericentrin. (TIF) [file pone.0148678.s005.tif]

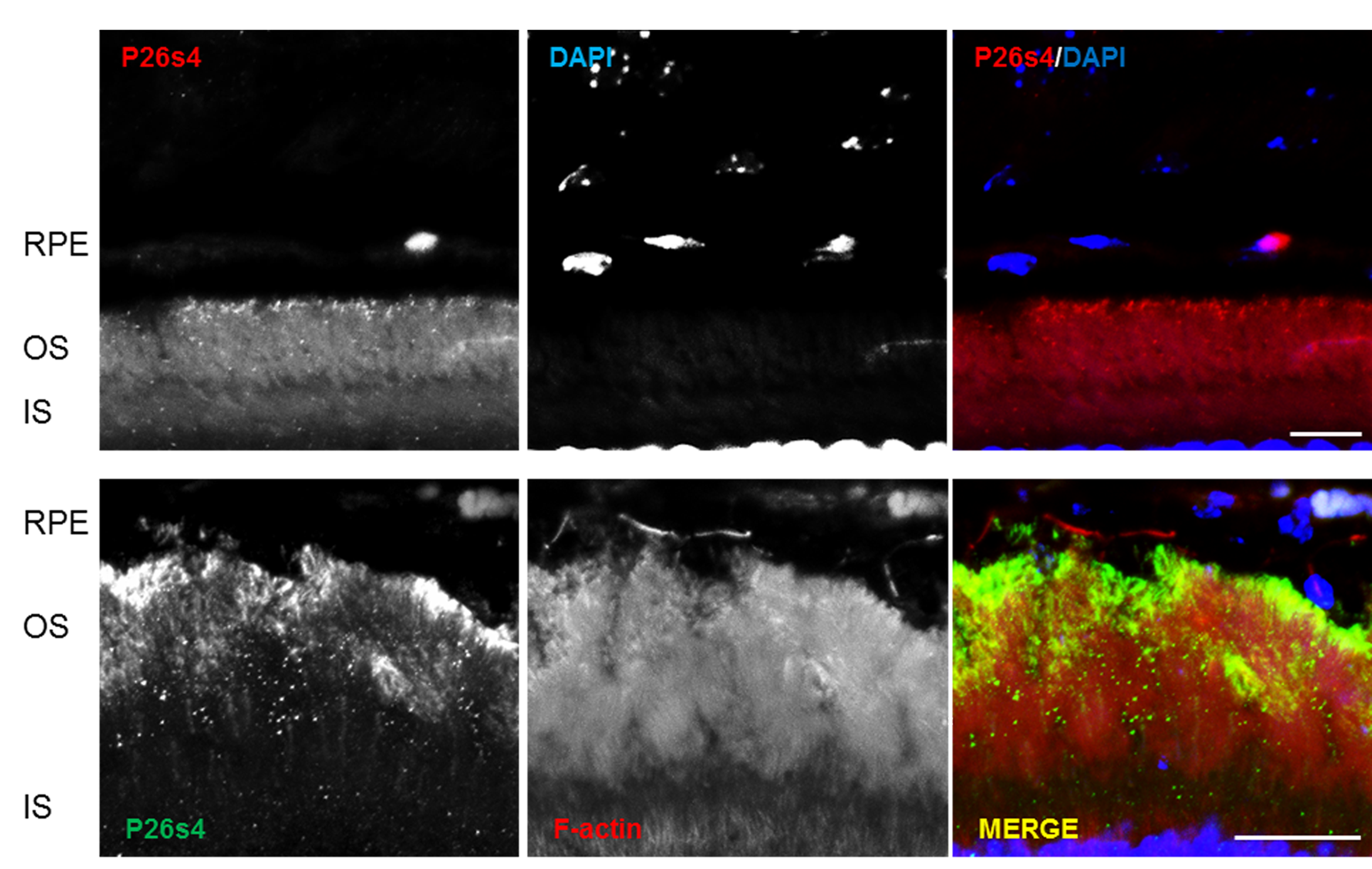

Supplement: S6 Fig — P26s4 localises diffusely throughout the photoreceptor OS with distinct P26s4 signal being observed at the RPE-OS interface and the RPE (both panels). Phalloidin staining for filamentous actin was used as a marker delineating OS structure (bottom panel). RPE, retinal pigment epithelium; OS, rod photoreceptor outer segment; IS, rod photoreceptor inner segment. Nuclei stained using DAPI. Scale bar 20 μm. Confocal microscope images were taken using the Zeiss LSM 700. Secondary antibody control images were collected using the same settings and generated no signals in the red and green channels. (TIF) [file pone.0148678.s006.tif]

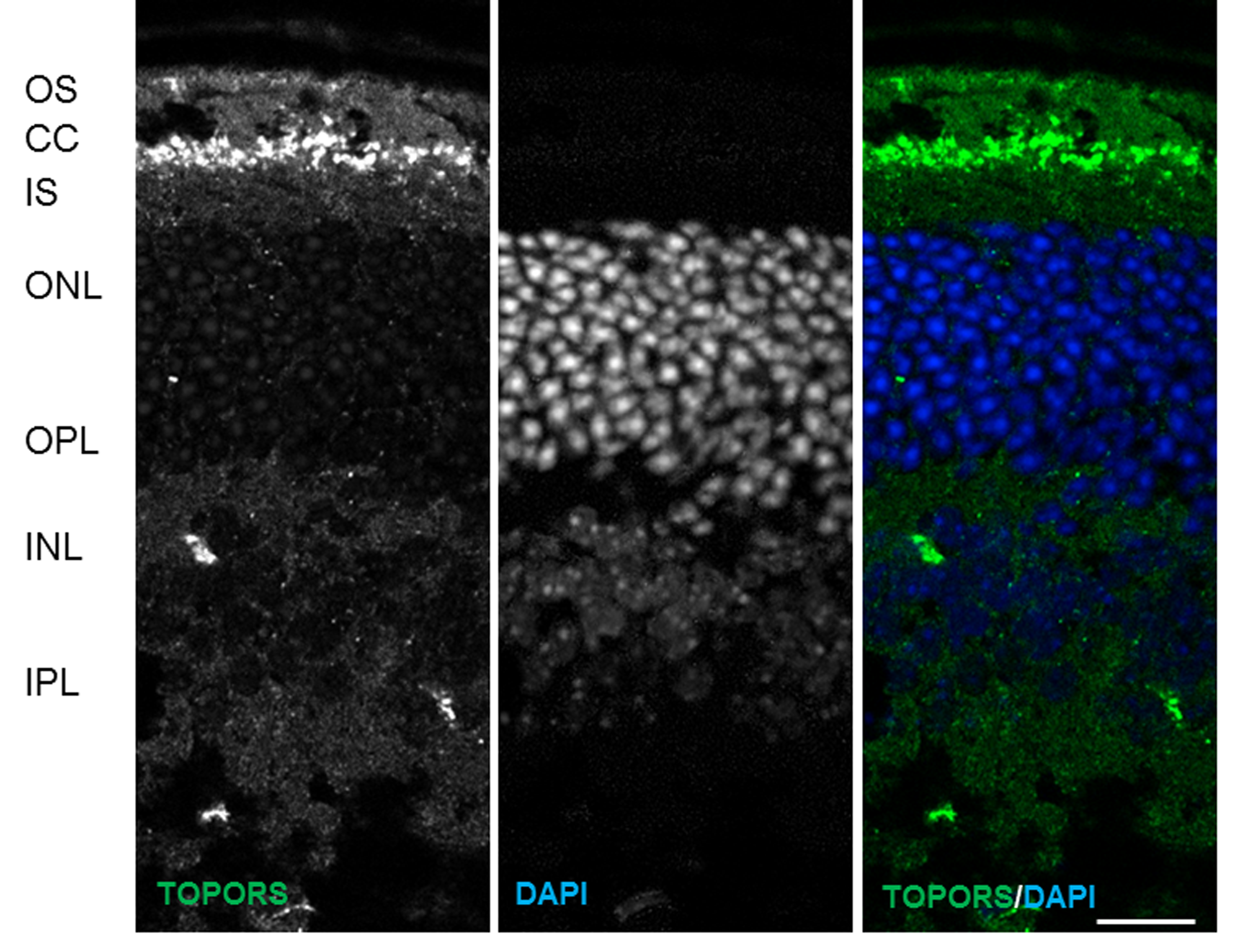

Supplement: S7 Fig — OS, photoreceptor outer segment; CC, connecting cilium; IS, photoreceptor inner segment; ONL, outer nuclear layer; OPL, outer plexiform layer; INL, inner nuclear layer; IPL, inner plexiform layer. Nuclei stained using DAPI. Scale bar 20 μm. Confocal microscope images were taken using the Zeiss LSM 700. Secondary antibody control images were collected using the same settings and generated no signals in the red and green channels. (TIF) [file pone.0148678.s007.tif]
